# Effects of recultivation on soil organic carbon sequestration in abandoned coal mining sites: a meta-analysis

**DOI:** 10.1038/s41598-022-22937-z

**Published:** 2022-11-22

**Authors:** Clara Baier, Antonia Modersohn, Friedrich Jalowy, Bruno Glaser, Arthur Gross

**Affiliations:** Institute of Agricultural and Nutritional Sciences, Soil Biogeochemistry, Martin-Luther-University Halle-Wittenberg, Halle (Saale), Germany

**Keywords:** Environmental impact, Carbon cycle

## Abstract

Opencast coal mining results in high loss of soil organic carbon (SOC), which may be restored via recultivation. Common strategies include liming, topsoil application, and phytoremediation. It remains unclear, however, which parameters determine the effectiveness of these varying recultivation strategies especially regarding SOC sequestration. This meta-analysis analyses the effect of varying recultivation strategies on SOC sequestration under different climate and soil conditions (pH, texture, depth) as well as in relation to time, based on 404 data entries from 51 studies. All included climatic regions recorded increases in SOC stocks, with tropical soils showing the highest potential for relative gains at up to 637%. We demonstrate that loamy soils sequester twice as much newly introduced SOC than sand. Strategy-wise, the highest mean rate of SOC sequestration is achieved by forest after topsoil application (3.9 Mg ha^−1^ a^−1^), agriculture after topsoil application (2.3 Mg ha^−1^ a^−1^), and agriculture with topsoil and fertiliser application (1.9 Mg ha^−1^ a^−1^) with a response ratio of 304%, 281%, and 218%, respectively. Soils analysed to less then 40 cm depth show higher SOC sequestration rates (< 10 cm: 0.6 Mg ha^−1^ a^−1^, < 20 cm: 1.0 Mg ha^−1^ a^−1^, and 20–40 cm: 0.4 Mg ha^−1^ a^−1^; response ratio of 123%, 68%, and 73%, respectively) than those analysed to a depth of 41–80 cm (0.1 Mg ha^−1^ a^−1^; response ratio of 6%). In terms of pH, strongly acidic soils (pH < 4.5) and alkaline conditions (pH > 7) offer the most beneficial environment for SOC sequestration at 0.4 Mg ha^−1^ a^−1^ and 0.8 Mg ha^−1^ a^−1^, respectively (185% and 273% response). Given comparable SOC sequestration potentials of forest after topsoil application, agriculture without amendments, and forest without amendments, we recommend to weigh these strategies against each other. Potentially decisive aspects are short- vs. long-term economic gains, food security concerns, and—in case of agriculture—the risk of overintensification leading to losses in SOC. Our data suggests that amendments exert considerable influence on SOC sequestration and need to be introduced under careful consideration.

## Introduction

Soils constitute the largest terrestrial carbon (C) pool (~ 1500–2400 Pg C), containing more than the combined amount of C retained in the atmosphere and biosphere^1^. Consequently, even the release of a small fraction of the soil-bound C into the atmosphere may elevate the level of atmospheric CO_2_ and, thereby, accelerate global warming^1^. A major driver of C loss from soils is human land cover change^2^, prominent and impactful forms of which are deforestation, agriculture, and surface mining. Presenting one of the first assessments based on satellite observations of gross forest cover loss combined with a map of forest C stocks, Harris et al.^3^ estimated that the net C emissions from tropical deforestation between the years 2000 and 2005 amounted to 0.81 Pg C a^−1^. A more recent analysis by Gatti et al.^4^ demonstrates that Amazonia, which holds approximately 123 ± 23 Pg C in total biomass and has been known as an important C sink, has turned into a net C source with a net biome exchange C balance of − 0.12 ± 0.40 Pg C a^−1^. The main drivers of this development are increasing deforestation and the intensification of the dry season induced by global warming, both of which increase the vulnerability of the forests to fire—a further driver of forest degradation^4^. Some authors, such as Sanderman et al.^5^, refer to this imbalance as a *carbon debt*; their estimate of the global C loss from the top 2 m of soil since the onset of agriculture suggests a total of 133 Pg C.

Mining for coal and other soil-based resources represents a particularly abrupt and disruptive land use change, with extensive off-site impacts that tend to expand over time (“spill-over effects”). An example is off-site deforestation to produce charcoal needed for iron ore processing and steelmaking^6^. At the same time, many coal reserves are located under forest covers^7–9^; in this light, it is unsurprising that mining (coal and other resources) caused 7% of the total forest loss of 46 tropical and subtropical countries in the year 2020 alone^10^. Where forest—especially tropical rainforest—is cleared to access the coal reserves, this coal’s negative climate impact results not only from burning it, but also from the loss of forests in their function as a C sink^11^. In addition to the C loss associated with deforestation and the effects of erosion, another mining-related practice that constitutes an important influence factor is topsoil removal. Common practice involves that the removed topsoil is stored for later re-application^12,13^. Depending on the extent of disturbance and the storage management, it may be exposed to soil quality-reducing processes during this period^13–18^. For instance, stockpiles exceeding the recommended height of 2 m lead to the formation of an anaerobic zone, and failure to establish a permanent vegetation impairs soil fertility through various mechanisms^13^. These mechanisms include soil compaction, reduced cation exchange capacity, reduced plant nutrient availability^15^, and negative effects on species composition and abundance of the microbial community^15,17,19,20^ as well as a decline in seedling emergence of indigenous plant species after re-application^12^. Some authors report losses in SOC of stockpiled topsoil that rise with increasing duration of storage (e.g.^15,21^). Such estimates, however, may be deceptive, as the reduced SOC content can simply originate from dilution (i. e. mixing of surface soils containing higher levels of SOC with deeper layers that are naturally lower in SOC)^22^. The mechanisms by which surface mining severely depletes SOC in both stored topsoil and the area of the pit itself include intensified erosion, altered temperature and soil moisture regimes, and reduction in organic matter returned to the soil^23^—leading, for instance, to enhanced mineralisation and leaching^23,24^.

Mine soils are anthrosols—that is, soils that have been altered profoundly by human activities^25^—classified as such when a surface mining operation permanently ceases production^26^. They are usually poor in nutrients and typically contain elevated levels of toxic elements such as Cu, S, As, Cd, Cr, Ni, Pb, and Zn, largely restricting immediate utilisation and posing a hazard to the natural environment as well as to human health^27^. Worldwide, a total area of 57,277 km^2^ is directly used for or affected by mining^28^, which equals approximately 0.04% of the global land surface area as given in Winkler et al.^29^. While mineral extraction activities are highly concentrated in only five countries—the mining industries of China, Australia, the United States, Russia, and Chile account for 51% of the globally mined area—the geographical distribution of areas under mining also reveals that very few countries are entirely inactive in this field^28^.

Particularly in the field of surface coal extraction, the scope of abandoned areas can be expected to increase in the near future. In the Global Coal to Clean Power Statement^30^ issued as a result of the COP 26 UN Climate Change Conference 2021, 46 countries acknowledge “that coal power generation is the single biggest cause of global temperature increases” and commit to the transition away from coal power generation. This shift is intended to be achieved on a global level by the 2040s^30^. Although this pledge is not supported by some of the worlds’ largest coal producers such as China, USA, Australia, and India^30,31^, the signatories include five of the world’s top 20 coal power-using countries and at least 23 countries whose commitment was entirely new^32^. Several European countries have already implemented policies to reduce their reliance on coal-fired electricity provision or have defined specific timetables for phasing out coal (“coal-exit”). This involves decommissioning lignite and hard coal power plants prior to the end of their technical lifespan, which is pursued by Denmark, France, the Netherlands, the UK^33^, and Germany^34^. Germany, for instance, officially aims to phase out coal “ideally” by 2030^34^. In contrast to some of the distant consequences of climate change, many of the negative impacts of coal use are local and near term (intragenerational) and may, therefore, be featured prominently in political energy strategy considerations^35^. In light of this development, recultivation of newly freed coal mining sites is going to gain in relevance and calls for comparative evaluation of different approaches. Historically, the term *recultivation* has been applied to different concepts and is not consistently used in scientific literature^36^. Out of the varying meanings given by Ignatyeva et al.^36^, this study defines recultivation as a collective term for a variety of measures designed to restore landscape ecosystems that have been significantly impaired by anthropogenic activities with the purpose of economic gain. The main objective of these measures is the restoration of ecosystem services that specifically qualify for human utilisation—i.e. strictly benefit-oriented, clearly distinguishing recultivation from renaturation (also referred to as restoration, revitalisation, or remediation). Once successfully restored, utilisation of these landscapes varies and includes agriculture, livestock farming, forestry, fishing, and recreational purposes. Of particular significance regarding global climate change is the effectiveness of different recultivation methods in terms of long-term C storage in reclaimed mine soils. Severely degraded soils such as mine spoils offer a large C sequestration potential—one reason being that they commonly contain low SOC stocks^37,38^. This deficit means they could store considerable amounts of additional SOC^39^. Storage and long-term stabilisation of SOC in soils is commonly known as SOC sequestration. This process describes how C that would otherwise be emitted as CO_2_ is incorporated into soils and converted into a long-lived C pool^40,41^. SOC sequestration can not only be considered an efficient strategy to remove CO_2_ from the atmosphere, but also has positive effects on soil quality, thus improving ecosystem functions and services, food security, and the resilience to climate change^42–44^. Common strategies to restore degraded mine soils include backfilling with overburden materials, topsoiling, and restructuring the land to near-original landscape contours to then recreate a vegetation cover^13,26,45^.

This study aims to provide decisionmakers with scientifically substantiated management recommendations with respect to SOC sequestration through selected available recultivation technologies. Considering that coal mines are currently or will be decommissioned all around the globe, particular emphasis was placed on obtaining a comprehensive dataset that is applicable across a wide range of environments. In doing so, we build on earlier studies such as a review published by Vindušková and Frouz^46^ in 2013, who addressed similar research questions but only contemplated data from temperate sites and did not consider agricultural recultivation. In terms of studies that summarise findings gained across large study areas, we also identified a number of studies focussing on the entire USA or specific parts of it, such as the Appalachian coalfields (e.g.^47–49^). A recent meta-analysis by Allory et al.^50^ provides insight into the SOC dynamics of a wide range of technosols, including mine soils, but does not differentiate between different types of mining.

The aim of this meta-analysis, therefore, is to provide a quantification of the development of SOC stocks in soils of abandoned and then differently recultivated coal mining sites worldwide. To this end, the objective is to calculate (i) the response ratio of SOC stocks influenced by recultivation, (ii) the SOC stock difference, and (iii) the rate of SOC sequestration, all of which are entirely based on data extracted from peer-reviewed studies. The second objective consists in identifying unambiguous evidence as to the impact of a set of influencing factors. The results are, therefore, grouped and analysed by climate zone, soil properties, recultivation strategy, and the period of observation.

## Material and methods

### Data source, collection, and categorisation

**Figure 1:**
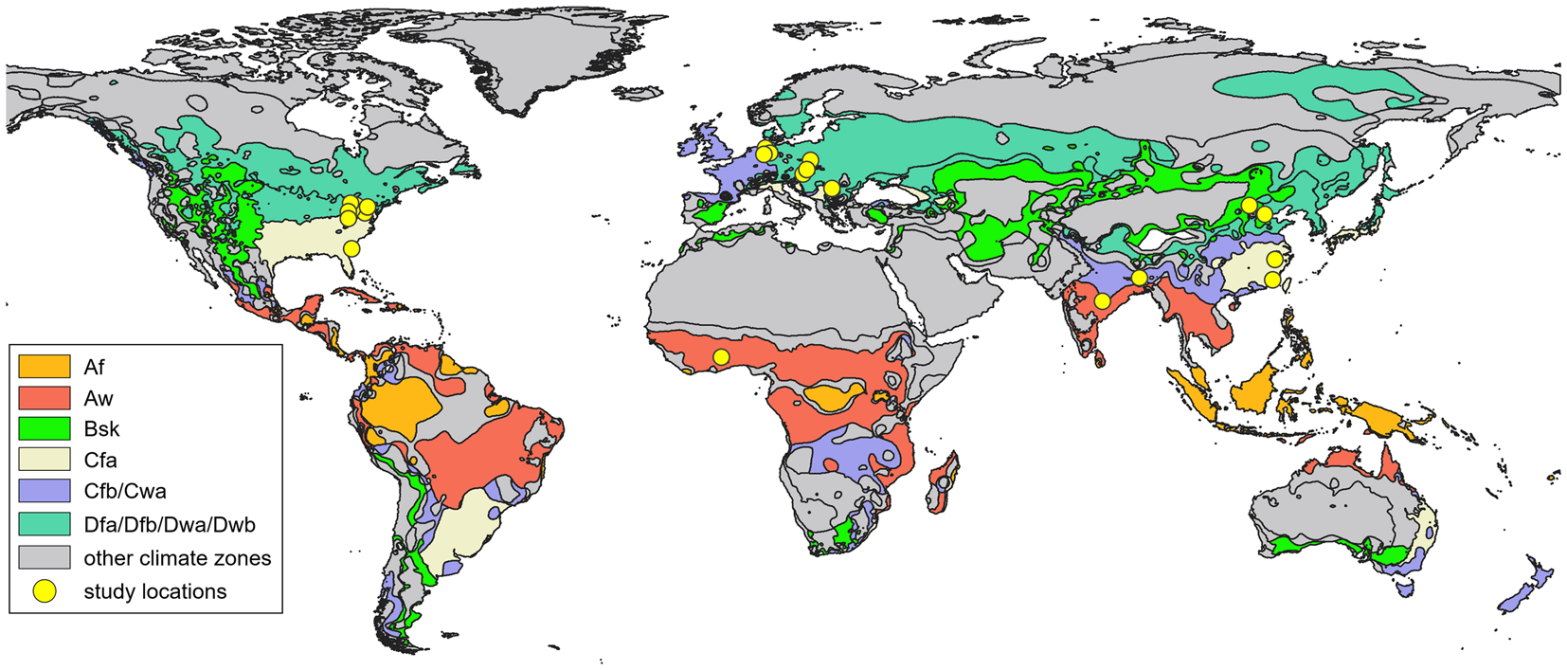
Global distribution of climate zones and study sites represented in this meta-analysis. The climate is classified according to Koeppen–Geiger (Af = tropical rainforest/equatorial climate; Aw = tropical savanna or tropical wet and dry climate; Bsk = semi-arid/semi-desert/steppe climate; Cfa = humid subtropical climate; Cfb/Cwa = oceanic/maritime/marine climate; Dfa/Dfb/Dwa/Dwb = humid continental climate). This map is based on climate zone data sourced from Peel et al.^53^.

Our analysis is based on a systematic literature query conducted in “ISI Web of Science (Core Database)” on February 4th, 2022, using the search string “mine (Topic) AND soil organic carbon (Topic)”. The search produced a total of 1,002 hits, all of which were evaluated as to their suitability for the purpose of this study in the following weeks. In total, 51 studies met our quality criteria (detailed in the following paragraph) and were included in our meta-analysis. These studies yielded an overall dataset of 404 pairwise (control and treatment) data entries which we used in our analysis. While our global search provided data from a variety of different continents and climates (Fig. 1), we were not able to identify any suitable studies from the Southern Hemisphere.

Studies were included in our analysis if they were performed under field conditions on an opencast mine and if effect and control sizes were quantified as either SOC or total organic carbon (TOC) stocks or expressed as content of SOC or TOC. If there were no control values given (which applies to 8 out of 51 studies), we used the SOC value after 1 year under recultivation as a control. This procedure was also applied where the provided control consisted of undisturbed soils under native forest/grassland ecosystems or agriculture unaffected by mining and located adjacent to or in the vicinity of the mining area (which applies to 5 out of 51 studies). We argue that while these values serve as a valuable baseline to define a target value or assess the effectiveness of recultivation in a specific setting (especially as a substitute where initial values are not available), they do not meet the quality requirements of an experimental control used to quantify and compare the exact response to different recultivation strategies across a variety of settings. A study by Ahirwal et al.^51^ clearly demonstrates that SOC sequestration in reclaimed mine soils behaves similarly to initial pedogenesis. This is further supported by Tan et al.^52^, who observed a minor increase in SOC content of 0.17% in the first year of coal mine recultivation, and also in agreement with the results of Bodlák et al.^52^. We, therefore, include studies that lack values at the beginning of reclamation but contain values after one year. A full list of studies, their number of observations, and the types of experimental controls included in this meta-analysis can be found in the supplementary material (Tab. S3).

Figure 1 provides a global overview of all climatic regions and study sites represented by this meta-analysis. For a high-resolution present-day map of climate zones as classified by Köppen-Geiger, see Beck et al.^54^. The number of studies represented in our analysis does not translate to the same number of sampling sites. An exact number of how many sites are included is not available, as some authors only provide the region their study was conducted in. In addition, some studies monitored more than one site. We can, however, discern several regional clusters that each housed several studies (e. g. the Rhenish and Lausitz lignite mining regions in Germany, the Ohio and Kentucky parts of the Appalachian Coalfields (USA), the Jharia coalfield in eastern India, or the Chinese province Shanxi).

In addition to information on the SOC content, we also extracted data on soil properties (initial SOC content, pH value, soil texture, bulk density), experiment characteristics (recultivation strategy, sampling depth, period of observation), and site characteristics (location, climate zone). In order to limit the diversity of the different soil texture classes, we grouped them according to the predominant grain size class (sand, silt, or clay). We added “loam” to represent the intermediate classes “clay loam” and “loam” using the triplet coordinate system of the World Reference Base^25^. If data was only presented in figures, the online tool Web Plot Digitizer Version 4.4^55^ allowed correct data extraction. If there was no direct information on SOC stocks given, we employed the following function provided by the FAO^56^ to derive the SOC stock:

1 $$SOC \; stock=SOC\times Bulk \; Density\times Depth\times 0.1$$

where bulk density is expressed in g cm^−3^, depth in cm, SOC in g kg^−1^, and 0.1 is a conversion factor to Mg ha^−1^.

In a few studies, no soil bulk density was given. In these cases, different pedotransfer functions were used. If the initial SOC, silt, and clay contents were provided, we used the pedotransfer function given in Men et al.^57^ (Eq. 2), where the silt and clay contents are expressed in %. If only information on initial SOC was provided, we used a pedotransfer function given in Manrique and Jones^58^:

2 $$Bulk \; Density=1.386-0.078\times SOC+0.001\times Silt+0.001\times Clay$$

3 $$Bulk \; Density=1.660-0.318\times {SOC}^{0.5}$$

This approach of estimating bulk density values to calculate SOC stocks was already applied in other meta-analyses^41,59^. Mine soils usually have comparatively high soil bulk densities (i.e. suffer from compaction) due to human influence, specifically the use of heavy machinery in combination with operations such as disturbance and backfilling^13,66,98^,^60^,^80^. To validate the use of pedotransfer functions that were intially developed for natural soils, we compared the measured with the calculated soil densities of our dataset. The mean of the measured densities is 1.45 g cm^−3^, while the mean of the calculated densities is 1.29 g cm^−3^ (see the supplementary material for a detailed graphic visualisation). This difference was considered satisfactorily low to justify applying functions 2 and 3 to our data. Although it admittedly leads to a slight systematic underestimation of SOC stocks, this causes considerably less inaccuracy than the alternative—eliminating all studies that do not report measured bulk densities.

For a better understanding of the factors influencing SOC stock changes, we grouped the changes in SOC reported by the studies by recultivation strategy, climate zone, soil texture, sampling depth, soil pH, duration of recultivation, and use of amendments such as mineral fertiliser or liming.

In total, we evaluated thirteen recultivation technologies within the framework of the four major strategy types “agriculture”, “agroforestry”, “forest”, and “topsoil application only”. Notably, the category “agriculture” comprises pasture, rotating crop systems, and monocultures. For a detailed list of individual subcategories within these strategies, see the supplementary material. Climate was classified according to Köppen-Geiger^53,54^, grouping similar (sub-)climates into the following categories: (i) Aw (tropical savanna or wet and dry climate), (ii) Af (tropical rainforest/equatorial climate), (iii) Bsk (semi-arid/semi-desert/steppe climate), (iv) Cfa (humid subtropical climate), (v) Cfb/Cwa (oceanic/maritime/marine climate), and (vi) Dfa/Dfb/Dwa/Dwb (humid continental climate). Each study site’s climatic attribution was determined using a 1 km resolution map (present-day version) provided by Beck et al.^54^. As for sampling depth, we created five classes to structure the strongly varying methodologies employed: < 10 cm, < 20 cm, 21–40 cm, 41–80 cm and > 80 cm). If samples from the same study varied in depth but all fell within a single one of the aforementioned categories, we summed them up to one value and added the value to the respective class in question. If samples ranged between two categories, the category containing the higher number of samples was chosen. Moreover, we present the SOC stocks of the upper 20 cm in two categories that appear to overlap but in fact do not: < 10 cm (= 0–10 cm) and < 20 cm (0–20 cm). The reason for this approach is that a few studies only analysed the upper soil layer between 0 and 10 cm, whereas the majority of our data was recorded in 0–20 cm. Given the high variability of sequestration in the topsoil layer, we considered the 0–10 cm data to be a valuable contribution and incorporated the raw data provided by these studies^61–65^. However, samples recorded only in less than 10 cm could not be extrapolated, as this would lead to an overestimation of SOC stocks. At the same time, it would be invalid to divide samples taken in 0–20 cm in half and assign one part to 0–10 cm and the other part to 10–20 cm—hence the two categories. We also did not proportionally increase any other values.

To generate comparable values in all other categories, we determined the SOC stock values categorised by analysed soil depth using the following equation:

4 $$SOC \; stoc{k}_{analysed \; soil \; depth}=\frac{SOC \; stoc{k}_{sampling\; depth}}{sampling \; depth}\times categorised \; depth$$

In terms of soil pH, we used the values reported after recultivation (as opposed to the initial/control situation) and grouped them into four classes: < 4.5, > 4.5–6.0, > 6.0–7.0, and > 7.0. To gain a better understanding of how the experiments’ duration (time of observation) influences SOC change, we grouped the studies into five classes: 0–5, 6–10, 11–20, 21–40, and > 40 years.

### Data analysis

Two different indices were used to estimate the effect of recultivation on SOC stock changes—one for relative and one for absolute changes. Relative effects are represented by the response ratio $R$ (resulting from the mean of the recultivation strategy divided by the mean of the control group, i.e. SOC stock after coal mining), while absolute effects are quantified by calculating the mean difference in SOC stocks (*ΔSOC*). Two similar absolute SOC changes can be the result of either a low or a high relative SOC change, depending on the initial SOC content and vice versa. Hence, to avoid erroneous conclusions, we calculated both indices.

*R* was determined using the following equation:

5 $$\mathrm{ln}(R)=ln\left(\frac{{X}_{E}}{{X}_{C}}\right)$$

where $\mathrm{ln}(R)$ is the natural logarithm of the response ratio $R$, *X*_*E*_ is the mean SOC stock of recultivated soil, and *X*_*C*_ is the mean SOC stock after mining (control group). Given that ratios generally have poor statistical properties^66^, we applied natural logarithm transformation to the response ratio to attain more appropriate statistical properties such as a symmetric distribution. To enhance the readability and for the purpose of interpretation and presenting the results in graphs, natural log-transformed data were back-transformed from ln(R) into R (R = e^ln(R)^).

We consistently converted the response ratio to a percentage (i. e. $R\times 100$) to present our results in a more intuitive dimension.

Statistical analyses were performed using R 4.1.0. We tested for normal distribution via Shapiro–Wilk Normality Test. The data was not normally distributed ($p<2.2\times {10}^{-16}$) and, as a result, log-transformed. Testing the log-transformed data for normal distribution using the Shapiro–Wilk-Test did not produce a valid result due to too many negative values. Outliers were identified and removed using the 95% confidence intervals. Given the lack of a normal distribution, we used Kruskal–Wallis-Test (α = 0.10) to establish whether there are significant differences between groups. Differences between individual samples were examined using the Dunn-Bonferroni-Test (α = 0.10).

As only a few of the analysed studies provide sufficient information on statistical measures and replicates, we applied an un-weighted meta-analysis to include as many strategy implementations as possible. Un-weighted meta-analyses—a common approach to quantify effects’ magnitudes^59,67–71^—assign all included studies the same weight (e.g. a weight of 1) as opposed to weighing larger studies more heavily than smaller ones^67^. To calculate the SOC stock difference, we used the following equation:

6 $$\Delta SOC={X}_{E}-{X}_{C}$$

where *X*_*E*_ represents the mean SOC stock in Mg ha^−1^ of the recultivated site and *X*_*C*_ the mean SOC stock in Mg ha^−1^ of the control site.

To comprehensively interpret the results, the 95% confidence intervals (*CI*) of the means of R and ΔSOC are calculated as follows:

7 $$CI \; upper=R \,or\,\Delta SOC*(1.96* \sigma/\sqrt{n})$$

8 $$CI \; lower=R \,or\,\Delta SOC*(-1.96* \sigma/\sqrt{n})$$

with the mean response ratio *R* or the SOC stock mean difference *ΔSOC* in Mg ha^−1^, the confidence coefficient being 1.96, σ the standard deviation, and *n* the number of individual strategy implementations.

Furthermore, we conducted a regression analysis to analyse whether the SOC sequestration rate and SOC stock difference are dependent on time after recultivation. As both indices indeed revealed significant dependence on time, we further investigated if there were significant differences between recultivation strategies and climate zones. The results of these tests are presented in the supplementary material.

Visualisation was realised using R Version 4.1.0^72^.

## Results and discussion

### Effect of recultivation strategy

**Figure 2:**
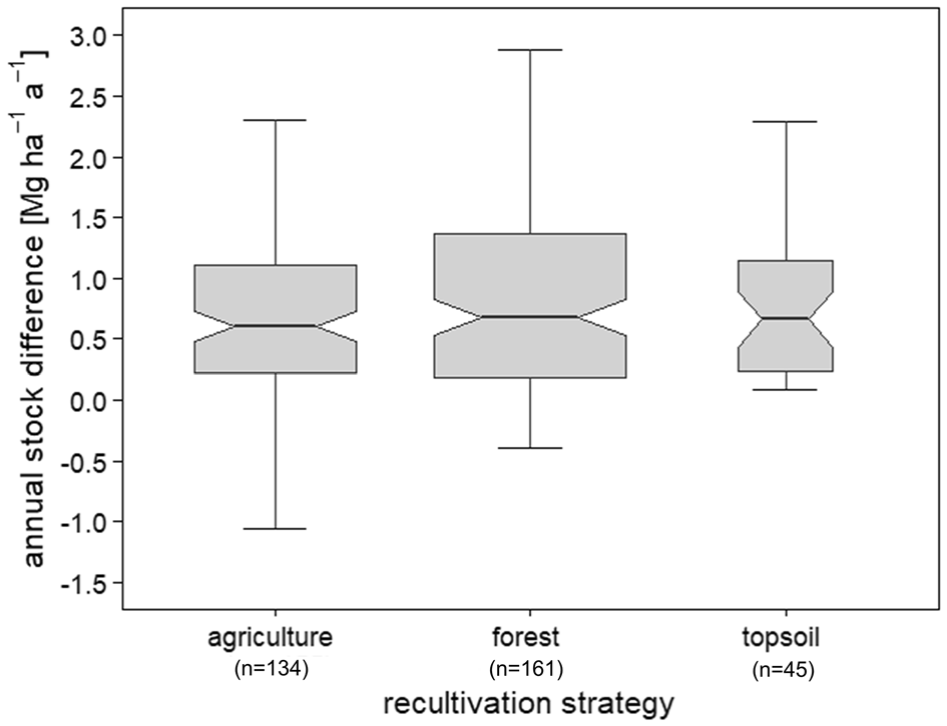
Annual amount of SOC sequestered by mine soils [Mg ha^−1^ a^−1^] that were recultivated employing the three main recultivation strategies (i) agriculture (incl. agriculture, agriculture with fertiliser, agriculture with topsoil, agriculture with topsoil and fertiliser, and agriculture with liming, topsoil, and fertiliser), (ii) forest (incl. forest, forest with topsoil, forest with liming, and forest with fertiliser), and (iii) topsoil (incl. topsoil by itself and topsoil with fertiliser). Agroforestry was omitted from this figure, as it was decidedly underrepresented with one single study containing 15 values. Each box contains the middle 50% of the data of a category. The mean of the data is shown as a horizontal solid line within the box. The whiskers indicate the lower and upper quartile of the data and are limited to 1.5 times the interquartile range. Outliers were removed from the boxplots in this figure to better visualise the differences between strategies. Notches represent the 95% confidence interval of the mean. The number of observations listed in the plot excludes outliers. The number of outliers that were removed is 21 for agriculture, 28 for forest, and 2 for topsoil. The original plot including all outliers can be found in the supplementary material.

**Figure 3:**
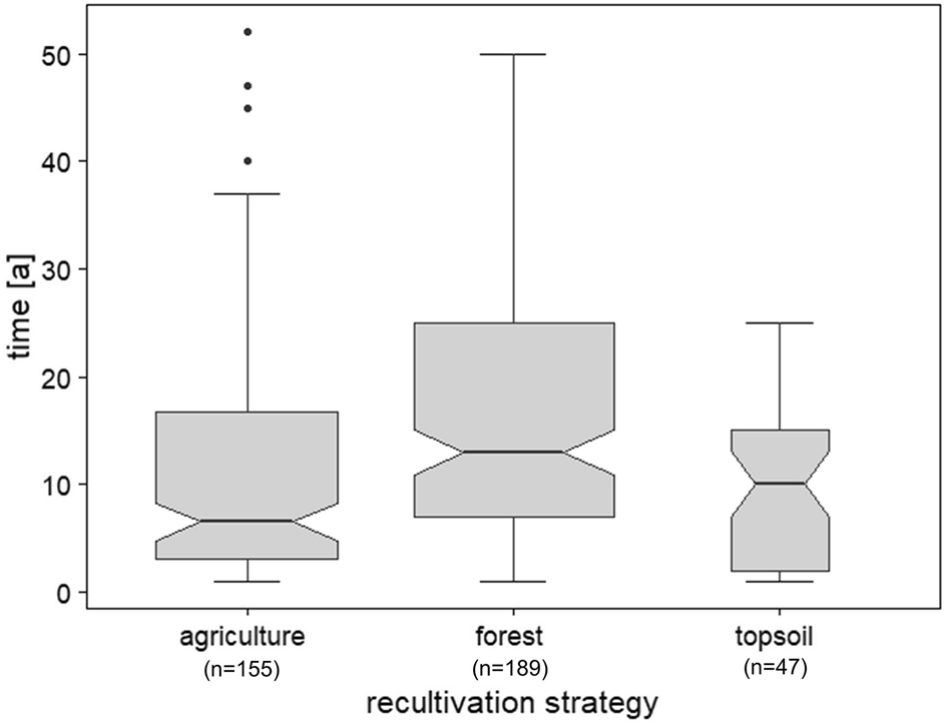
Time period covered by the entirety of individual observations that make up each of the three main strategies, respectively. Each box contains the middle 50% of the data of a category. The mean of the data is shown as a horizontal solid line within the box. The whiskers indicate the lower and upper quartile of the data, respectively, and are limited to 1.5 times the interquartile range. Notches represent the 95% confidence interval of the mean. Agroforestry was omitted from this figure, as all values within this category stem from one study and represent a single point in time.

Figure 2 shows that forest, agriculture, and sole topsoil application record rather uniform rates of mean annual SOC sequestration at 2.0 Mg ha^−1^ a^−1^, 1.7 Mg ha^−1^ a^−1^, and 1.2 Mg ha^−1^ a^−1^, respectively. Considering that we calculate the annual sequestration rate based on the total time under recultivation and the total amount of SOC sequestered over this period, the results of strategies covering different periods of time are combined in one category (Fig. 3). Temporal effects and the extent to which they may influence the success of the different recultivation strategies examined in this study is discussed in detail in the chapter “Temporal effects”.

**Figure 4:**
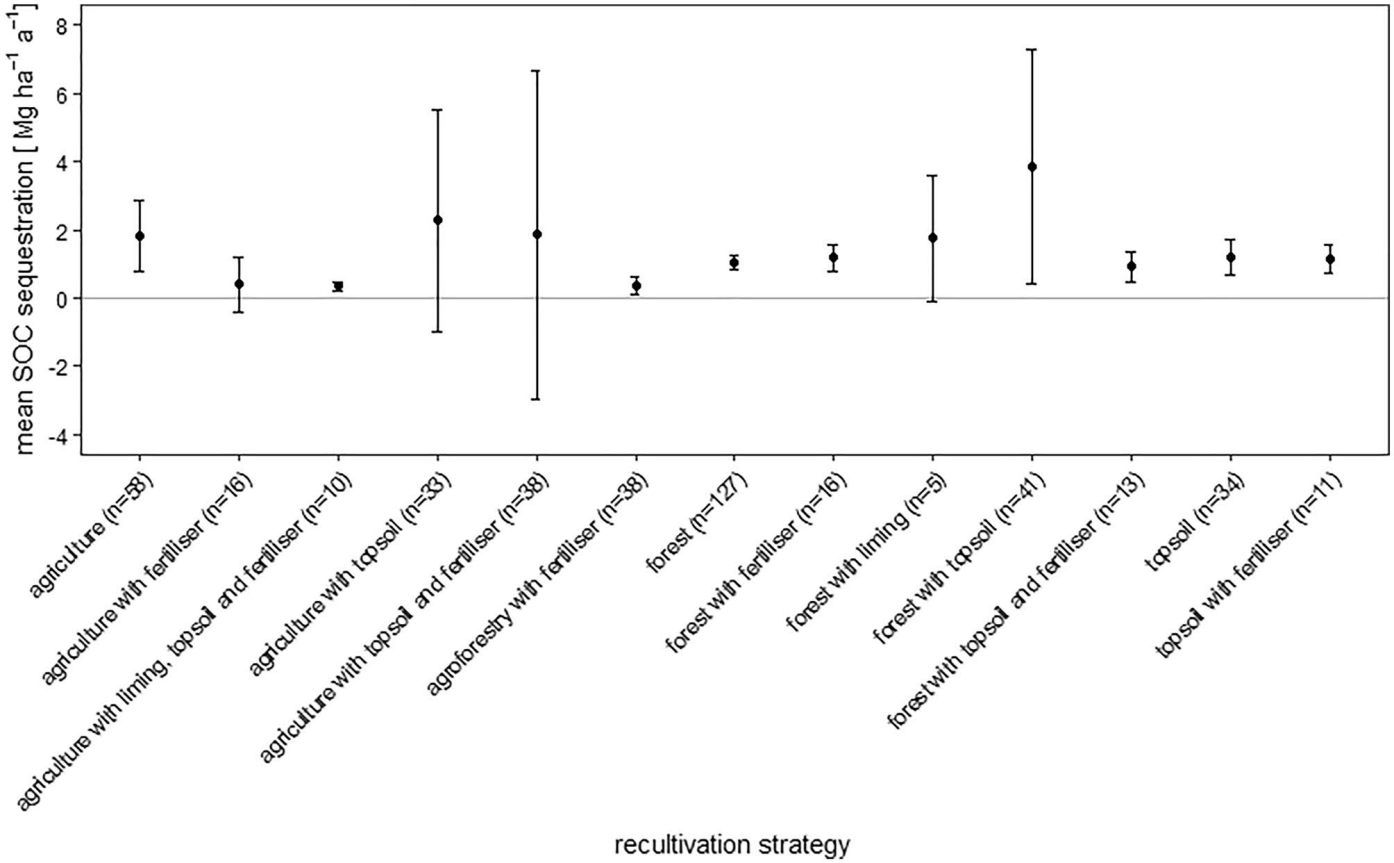
Mean annual SOC sequestration rate [Mg ha^−1^ a^−1^] of all 13 recultivation strategies.

In our study, the mean annual SOC sequestration rate of 2.0 Mg ha^−1^ a^−1^ recorded by forest is the highest one of the three main strategies (Fig. 2). The different annual sequestration rates within the overall category forest do not vary strongly apart from forest after topsoil application, the difference between the highest and lowest individual rates of the remaining subcategories amounting to 0.8 Mg ha^−1^ a^−1^ (Fig. 4). Comparing these results with a study by Lal^73^ that looked at forest soils outside the context of recultivation, our results are noticeably higher. The annual sequestration rate of forest soils ranged between 0.9 Mg ha^−1^ a^−1^ and 0.2 Mg ha^−1^ a^−1^^24^. Our results are derived from measurements that cover observation periods of up to 50 years. This puts the initially high storage capacity of afforestation into perspective. Akala and Lal^74^ reported a C storage potential of afforested mine soils of 2–3 Mg ha^−1^ a^−1^ in the first 20–30 years. After that, 0.4–0.7 Mg ha^−1^ a^−1^ were stored^74^.

At 0.4 Mg ha^−1^ a^−1^, the mean rate of annual SOC sequestration achieved by agroforestry falls short of those of agriculture, forest, and fallow after topsoil application. It is, however, based on only 15 pairwise data comparisons from a single study of an agrosilvicultural system conducted under summer-warm humid continental climate (Dfb). Considering that these measurements were also all gathered within the same year, agroforestry is distinctly underrepresented in our meta-analysis and does not allow for sufficiently substantiated conclusions. This lack of data calls for further research as to the specific effects of agroforestry on SOC sequestration in former coal mining environments.

**Figure 5:**
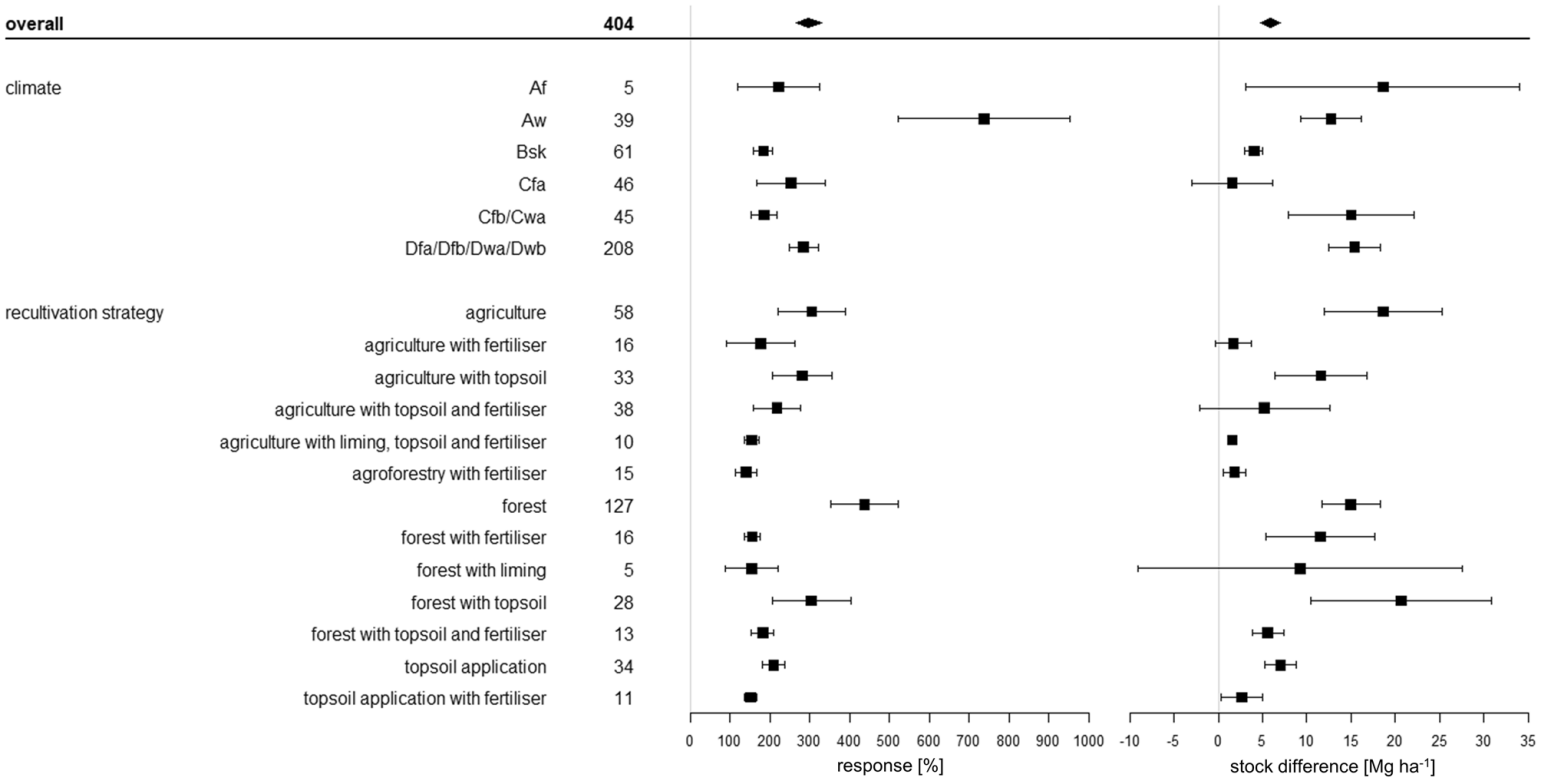
Relative response of soil organic carbon stocks [%] and absolute difference in soil organic carbon stocks [Mg ha^−1^] influenced by thirteen different recultivation strategies and climate. The climate is classified according to Koeppen–Geiger (Af = tropical rainforest/equatorial climate; Aw = tropical savanna or tropical wet and dry climate; Bsk = semi-arid/semi-desert/steppe climate; Cfa = humid subtropical climate; Cfb/Cwa = oceanic/maritime/marine climate; Dfa/Dfb/Dwa/Dwb = humid continental climate). The overall grand mean of all individual strategies is presented in the first row, whereas the values below correspond to the individual categories. Each response ratio is presented as the mean with the upper and lower 95% confidence intervals. The confidence interval of the grand mean is visualised by the solid square. The number in each strategy row expresses on how many pairwise comparisons the statistics were based.

The diversity of management options included in the category agriculture (pasture, rotating crop systems, and monocultures) may explain the relatively wide range of SOC sequestration rates (Fig. 4). Fertiliser application, specifically compost expanded with bentonite and mineral fertiliser (N, P, K, Mg), can have a negative effect on SOC sequestration (Fig. 5). The mean SOC sequestration rate under agriculture determined in this study amounts to about 1.7 Mg ha^−1^ a^−1^. Our results indicate that topsoil application increases the capacity to sequester SOC by agricultural recultivation (Figs. 4 and 5), which is in agreement with previous findings by Shukla and Lal^75^, who demonstrated that topsoil application increases the rate of SOC sequestration and, importantly, develops the greatest positive effect when combined with agricultural recultivation. As for pasture soils, previous long-term sampling after 25 years of recultivation has demonstrated high SOC sequestration capacities^74^, which is another aspect to be considered in relation to the comparatively large span of SOC stock gains achieved by agriculture (Figs. 4 and 5). In fact, the high SOC gains achieved by pastoral farming represent the upper end of this span, which highlights the SOC sequestration potential of comparatively extensive forms of agriculture.

In terms of absolute SOC stock differences, some recultivation strategies are clearly above the range of the grand mean (11.85 Mg ha^−1^), while others range below (Fig. 5). All strategies show both absolute and relative positive effects. The strategies “agriculture”, “agriculture with topsoil”, and “agriculture with topsoil and fertiliser” increase SOC stocks by 18.7, 11.6, and 5.2 Mg ha^−1^, respectively. The highest relative response of 338% is achieved by forest without any additional amendments. The strategy “forest with topsoil” shows the largest absolute effects on SOC stocks with 20.7 Mg ha^−1^. The greatest positive response of SOC sequestration rates and promoted SOC sequestration is achieved by the addition of topsoil combined with the establishment of agriculture and afforestation (Fig. 5). The strategies “topsoil application” and “topsoil application with fertiliser” result in high response ratios of 210% and 151%, respectively, but only register a relatively small absolute increase in SOC in the case of “topsoil application with fertiliser” of 2.7 Mg ha^−1^. Similarly, the addition of fertiliser to agriculture only results in a slight increase of 1.7 Mg ha^−1^, yet corresponds to 177% relative response. Notably, the addition of amendments like fertiliser (11.6 Mg ha^−1^, 156%), liming (9.3 Mg ha^−1^, 155%) or a combination of topsoil and fertiliser (5.6 Mg ha^−1^, 182%) to forest lead to distinctly lower SOC stock differences than those achieved by forest without any additional amendments (15.0 Mg ha^−1^, 438%).

The small increase in SOC in the categories “agriculture with fertiliser” and “agriculture with liming, topsoil, and fertiliser” can mainly be explained by the intensive agricultural use with short-term crop rotation and loss of nutrients. Rodionov et al.^50^ noted that the beneficial effect of fertiliser amendment—i.e. enhanced crop growth—was only pronounced in monoculture settings. They found no detectable effect by an additional organic matter input through crop residues combined with digester compost. Li et al.^75^ showed that fertilisation amendments increased the total SOC in the surface layer. In case of agriculture, the risk of overintensification leading to SOC losses should always be considered before choosing a recultivation strategy and when managing agricultural systems.

As for topsoil, Fig. 5 clearly shows that the addition of topsoil to forest can increase SOC sequestration. This is consistent with the findings of Akala and Lal^74^, who demonstrated that mining areas reclaimed with topsoil can sequester more SOC than those without topsoil application. Their direct comparison of forest with and without topsoil application revealed that forest without topsoil application stores more SOC at the beginning of the recultivation period. While this reflects the general trend over time, the annual rate of SOC sequestration showed notable fluctuations over time. The authors attribute the variability of SOC sequestration in different years to fluctuations in the nitrogen balance^74^. The results of our analysis are in agreement with the conclusion reached by Akala and Lal^74^—specifically, that coal mine recultivation using topsoil application may generally achieve more SOC sequestration than strategies without topsoil application^74^.

Our results further show that the application of topsoil has positive effects on soils under both agricultural and silvicultural recultivation of coal mines: The relative and absolute development of SOC stocks as well as the annual sequestration rates are positively influenced.

### Climate effects

All five climatic regions studied show both a positive relative and absolute SOC increase (Fig. 5) after recultivation. The response ratio (i. e. relative effect) ranged from 184% (Bsk, semi-arid/semi-desert/steppe climate) to 737% (Aw, tropical savanna or tropical wet and dry climate) (Fig. 5). Absolute SOC stock differences range between 1.6 Mg ha^−1^ (Cfa, humid subtropical climate) and 18.6 Mg ha^−1^ (Af, tropical rainforest/equatorial climate) (Fig. 5). The high response of SOC stocks to recultivation observed in tropical rainforest/equatorial climate (Af) poses by far the most prominent deviation from the grand mean and clearly stands out against the distinctly lower, nearly uniform responses recorded under all the other climates, the difference to the second highest response (recorded under Af climate) being 452% (Fig. 5). The absolute differences in SOC sequestration under different climates are much more heterogeneous than the relative responses (Fig. 5).

Climatic conditions—specifically temperature and precipitation—are generally considered to be a key, if not the most important influence factor on SOC dynamics, and they explain the majority of variation observed in SOC turnover (and C input into soils) on both a global and (sub-)regional level^76,77^. In addition to precipitation controlling the plant net primary production and, consequently, the amount of vegetation-derived C supplied to the soil, moist soil conditions promote the formation of weathered mineral surfaces that act as SOC-stabilisers^77^. Furthermore, high soil water contents are often associated with acidity^77^, which restricts microbial SOC decomposition^77,78^. Microbial C cycling, however, is also a highly sensitive to temperature^77^. A review by Wiesmeier et al.^77^ identified numerous studies indicating a general decrease of SOC with rising temperatures, which leads to the occurrence of highest SOC stocks in cool humid environments and correspondingly lower reserves in warmer, drier climates. These relationships are confirmed by Carvalhais et al.^79^, who revealed a clear dependence of C turnover times at terrestrial ecosystem level on both temperature and precipitation.

In this context, the high relative response of 737% our study registered in tropical savanna or tropical dry and wet climate (Aw) can be explained by the fact that the initial SOC content and stocks are comparatively low in the control sites. In this setting, even small absolute increases in SOC sequestration in soils with low initial SOC can lead to large relative gains^59^. Similar effects were observed by Ivezić et al.^80^ in a meta-analysis of SOC dynamics under agroforestry systems. The authors report that the desired sequestration effects were higher in tropical regions characterised by comparatively low initial SOC contents than in temperate areas with soils rich in SOC^80^. It has to be highlighted, however, that these developments need to be monitored over time: A meta-analysis by Ma et al.^81^ found that while agroforestry systems in tropical regions were able to quickly increase SOC to peak levels, SOC in temperate agroforestry systems recorded a slower rate of SOC sequestration but peaked at a greater level of SOC. In our study, in addition to only covering a span of 2–9 years, the Af climate value is only based on 5 pairwise readings and, therefore, markedly underrepresented in comparison to all other regions. The Aw value, while based on more observations (n = 39), also only represents only 2–13 years of monitoring. This may, to some extent, account for the pattern shown by Fig. 5.

### Temporal effects

**Figure 6:**
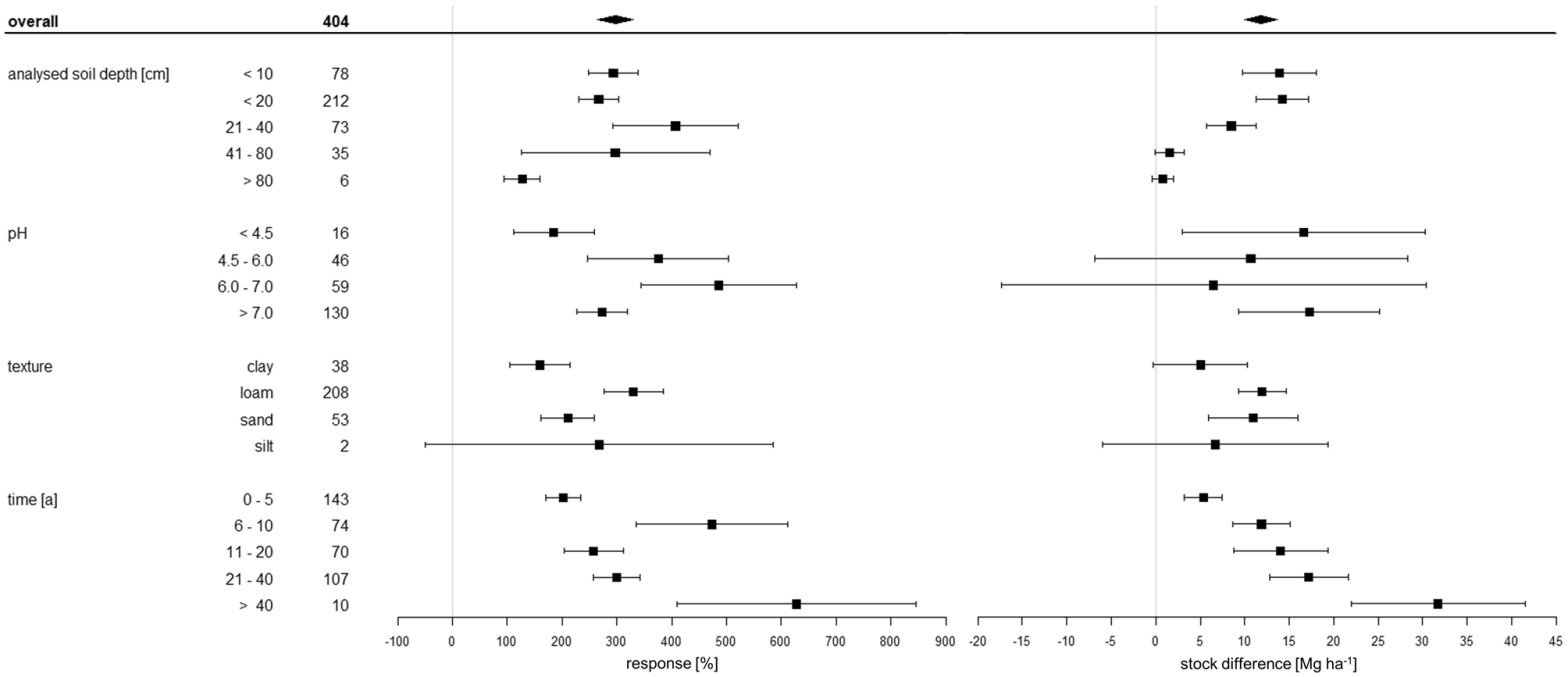
Relative response of soil organic carbon stocks [%] and absolute difference in soil organic carbon stocks [Mg ha^−1^] influenced by analysed soil depth [cm], soil pH, soil texture, and time under recultivation [a]. The overall grand mean of all individual strategies is presented in the first row, whereas the values below correspond to the individual categories. Each response ratio is presented as the mean with the upper and lower 95% confidence intervals. The confidence interval of the grand mean is visualised by the solid square. The number in each strategy row expresses on how many pairwise comparisons the statistics were based.

The absolute SOC stock differences show a continuous pattern of increase with rising time under recultivation. Moderate values of 5.4 Mg ha^−1^ SOC in the early stage (0–5 years) increase to 31.7 Mg ha^−1^ SOC after more than 40 years of recultivation (Fig. 6). Sites where recultivation was initiated more than 40 years ago also show the second largest relative effect of all investigated temporal classes at 627% (Fig. 6). At the same time, this category draws on the lowest number of pairwise observations (n = 10) with the longest period of recultivation covered being 52 years. The prominent effect that is nonetheless achieved in these studies highlights the need for more long-term experiments in different environments and with different experimental strategies to substantiate the impression generated by the temporal development presented in Fig. 6. Six out of the ten observations with a duration of more than 40 years are observed in the recultivation category forest, the overall insight being that mature forests display larger SOC pools than juvenile stands—a result that is conform to earlier observations of studies conducted within and outside the context of recultivation^74,82,83^. A meta-analysis of SOC stock dynamics following afforestation in Northern Europe by Bárcena et al.^83^ revealed that overall SOC stocks increased with forest age, although only the 0–20/0–30 cm soil layer exhibited a statistically significant increase at 0.4% a^−1^. Crucially, the findings indicated that the direction of change in SOC stocks is strongly variable during the first 30 years after afforestation^83^. Later stages, however, were found to be consistently characterised by gains in SOC stocks^83^. As our study evaluated SOC stock changes in the periods of 21–49 years and > 40 years following recultivation, this 30-year threshold identified by Bárcena et al.^83^ may be more pronounced than the results suggest. The overall trend, however, is consistent with their findings, given the distinct increase in SOC sequestration in soils that have been subjected to recultivation for more than 40 years compared to shorter periods (albeit, notably, these results also include data from recultivation strategies other than afforestation). A model prediction by Akala and Lal^74^ proposes that recultivation via forest establishment after topsoil application may need 100 to 150 years for the SOC pool to achieve a state of equilibrium (i. e. levels that are natural and/or achievable at the site in question). A very important factor, especially in coniferous forests, is the contribution of forest floors to SOC stock change^82–84^. Bradford and Kastendick^82^ found the C content in forest floor material to increase linearly with stand age in pine forests in the Northern USA.

The aging effects of SOC sequestration in grassland (represented by 15 studies within the category “agriculture”) are no less pronounced and relevant than those of forests. Nakagami et al.^85^ investigated the soil C stocks of the upper 25 cm in 24 Japanese pasture grasslands aged 3–43 years, 14 of which were compared to the soil C stocks of adjacent forests. The analysis showed highly similar values between both vegetation types, suggesting grassland to be just as potent an option for long-term C sequestration as forest. Čížková et al.^86^ compared the soil C stocks of reclaimed (seeded cultural grass-legume mix with topsoil application) and unreclaimed (levelled and spontaneously colonised by grassy vegetation) grassland sites formerly used for opencast lignite mining in the Czech Republic. Their chronosequence of up to 52 years under reclamation showed a significant increase in soil C stocks over all 12 sites and soil depths. Soil C sequestration of reclaimed sites, however, took place considerably faster than in unreclaimed sites^86^. The total C stock in 0–18 cm depth of 50-year-old reclaimed sites increased by approximately 8% since the onset of reclamation, which corresponds to a sequestration rate of 1.6 Mg C ha^−1^ a^−1^^86^. While young unreclaimed sites recorded a higher soil C content than young reclaimed sites, this relationship was reversed with age^86^. Akala and Lal^74^ present the model prediction that recultivation by grassland with topsoil application may achieve an equilibrium after 110 to 140 years of recultivation. At the same time, it is important to note that the influence of different management regimes on SOC sequestration may outweigh temporal effects in grasslands^87^. High-disturbance but infrequent interventions such as re-seeding (i.e. ploughing and seeding of grasslands with more productive grass cultivars) appear to impair the ability of grassland soils to sequester C significantly less than regularly employed management strategies such as livestock grazing and the use of heavy farm machinery. The reason is that the latter affect soil compaction, and soil C stocks significantly decreased with increases in soil bulk density^87^.

Consequently, the factor time poses a strongly influential variable that must always be considered in combination with other aspects such as management practices. As most of the values in our analysis is only cover a period of 0–5 years (n = 143) and the category > 40 years is strongly underrepresented at n = 10, more long-term experiments with durations > 40 years are needed to quantify and substantiate the effect of time for all strategies.

### Soil depth effects

In terms of soil depth, considerably more SOC is stored in the upper 40 cm compared to areas deeper than 40 cm, both in relative and absolute terms (Fig. 6). The highest SOC sequestration is recorded at soil depths from 0 to 20 cm, with a mean stock increase of 14.3 Mg ha^−1^ for 212 pairwise comparisons. The uppermost range < 10 cm shows similar absolute and relative increases in SOC at < 20 cm with 13.9 Mg ha^−1^ and 293%. Here it is also noticeable that the values have a greater dispersion and exceed the < 20 cm category in both directions with 9.7 Mg ha^−1^ lower CI and 18.0 Mg ha^−1^ upper CI. The 21–40 cm layer shows the highest response at 407%, with a mean SOC increase of 8.5 Mg ha^−1^ for 73 pairwise comparisons. Keeping up with this trend of decreasing sequestration with growing depth, comparatively modest gains of 1.52 Mg ha^−1^ (297%) are observed at 41–80 cm depth. The deepest area shows an even smaller increase of 0.8 Mg ha^−1^ (127%). The last categories show negative values in the lower CI, relative and absolute, and thus SOC losses.

Influences that are often reported to diminish with decreasing depth are the content of organic matter, the number of microorganisms, and biological activity^88^. These aspects may be responsible for the decreasing SOC content from the upper soil layers to the lower layers as displayed in Fig. 6. Especially in forest soils, this plays an important role^89^. Another influential factor can be soil management. For example, no-till management on agricultural land leads to SOC stabilisation in the surface soil layer (< 10 cm)^89^. We find the second highest SOC stock difference in the < 10 cm layer. This near-surface management-dependent C stabilisation can be reversed with a change in management^90^. The pronounced relative effect in the 21–40 cm range with an absolute increase of 8.5 Mg ha^−1^ is highly important for long-term C storage^90^ and may be a result of varying factors. One possible explanation is that this manifestation could be related to the coarse-textured condition of recultivated soils. Under these circumstances, the combination of topsoil application, fertilisation, and recultivation could result in greater transport of organic C, which then translocates to the 21–40 cm soil layers^75,89^. Angers and Eriksen-Hamel^89^ observed that if topsoil is applied prior to recultivation, its displacement through the coarse pores of the dump substrate can lead to higher sequestration. Specifically, SOC shifts to deeper soil layers in these systems, which can be termed convective. Furthermore, this depth range (21–40 cm) in our study also contains 31 pairwise comparison with the strategy “forest”. Due to the largely unobstructed, deep lateral root spreading and an increase in fine root development of trees on recultivated areas^87^, the tipping substrate is loosened up and SOC is also enriched in depths of 21–40 cm. SOC is thus transferred to the subsoil, i.e. to areas where there is a high deficit between actual and potential sequestration capacity^75^. Another relevant factor could be the included studies in which farming systems were tilled. The reason is that in these systems, SOC usually accumulates just below the plough bottom, i.e. precisely in depths between 21 and 40 cm^86^. In addition, soil bulk density increases with increasing soil depth^51,62,88^. Mine soils usually have comparatively high soil bulk densities (i.e. suffer from compaction) due to human influence, specifically the use of heavy machinery in combination with operations such as disturbance and backfilling^13^,^66^,^98^,^60^,^80^. The addition of topsoil—especially if applied using low-compaction grading techniques^92^—can lower the bulk density, improving soil structure and ventilation/infiltration characteristics^13^. Further beneficial effects on soil bulk density can be achieved by establishing a vegetation cover, as plant root penetration has been found to be an important driver of soil aggregate stability in many settings^75^;^92^;^93^. Both topsoil addition and revegetation influence the amount and quality of soil organic matter content^13^,^75^,^94^, whose bonding properties promote soil aggregation, hence increasing porosity and, thereby, lowering soil bulk density^94^.

### pH effects

The evaluation of pH effects on the difference in SOC stocks reveals a general tendency of poorer SOC sequestration potentials in neutral to near-neutral soil conditions. Alkaline soils at pH > 7 result in the highest absolute mean increase at 17.3 Mg ha^−1^, closely followed by the increase of 16.7 Mg ha^−1^ recorded in strongly acidic soils with a pH < 4.5 (Fig. 6). The lowest absolute but highest relative increase are 6.5 Mg ha^−1^ (486%) at pH 6.0–7.0 (Fig. 6). Overall, the differences in relative response are smaller than those of other influence factors such as analysed soil depth, texture, or time (Fig. 6). Importantly, it has to be noted that only about half of the studies contained information on the pH and our data on pH effects is characterised by large confidence intervals that overlap to a great extent, both of which limits the conclusiveness of our deductions.

The indications that moving away from neutral conditions towards either direction appears to be beneficial to SOC sequestration are consistent with previous findings. Several studies have linked changes in soil pH to the rate of C cycling in soils and investigated the underlying mechanisms of these dynamics with respect to the role of microbial activity^76,78,95–97^. There is growing evidence that physical and chemical soil properties—pH being an important component—have significant direct effects on SOC stability^76^. Specifically, they influence physico-chemical barriers that keep microorganisms from accessing soil C sources or directly control the microbial processes leading to SOC decomposition (specifically microbial enzyme activity and community composition)^76^. Malik et al.^78^ discerned a threshold above and below of which microbial C sequestration in soils takes place, but is a result of different mechanisms. They found that a land-use induced rise in pH above a threshold of ~ 6.2 causes SOC losses as a result of intensified decomposition processes which were previously inhibited by acid retardation of microbial growth. At pH > 6.2, however, SOC sequestration can also *originate from* stimulated activity (i.e. an efficient substrate metabolism) of certain microbes. At pH < 6.2—combined with wet conditions—, abiotic factors limit microbial growth and decomposition, thereby causing accumulation of SOC^78^. These dynamics may explain the pattern of highest absolute SOC stock increases below pH 4.5 and above pH 7.0 revealed by our analysis.

Malik et al., therefore, encourage to consider the given soil pH a key to designing land management strategies that are effective in enhancing SOC sequestration^78^. For near-neutral pH soils, they recommend less intensive management practices that increase beneficial microbial growth efficiency^78^. While mine soils normally have a low pH^13,98,99^, they have also been known to reflect the other extreme of strongly alkaline conditions^13^. The advice provided by Malik et al.^78^ for acidic soils involves intensifying plant production and prioritising the management of abiotic C-accumulating factors such as acidity and wetness^78^. This is consistent with the recultivation strategies considered in this meta-analysis, which target both soil pH and vegetation cover. The suitability of varying crop, grassland, or tree species for low vs. high pH regimes, however, is not considered and would be essential to a fully integrated recultivation strategy. The same is true for soil moisture, which is also not evaluated in this study (although it is partially implied by the investigation into climatic influences) and poses a subject for future consideration.

### Soil texture effects

Higher silt contents have a positive effect on SOC stocks with a high relative response ratio of 268% and an absolute mean increase of 6.7 Mg ha^−1^. This high response of silt, however, is based on only two values. Especially with silt, it is important to consider the range, because the two values are very far apart (between -6.0 Mg ha^−1^ and 19.4 Mg ha^−1^) and thus conclusive to a limited extent. Loam is found slightly above the range of the grand mean and shows the highest relative response of 330%—strongly exceeding that of sand (210%) and clay (160%). However, the absolute increases in loam and sand differ only slightly. At 12.0 Mg ha^−1^, loam exceeds sand (11.9 Mg ha^−1^). Sand covers a much wider range between the lowest (5.9 Mg ha^−1^) and highest (16.0 Mg ha^−1^) values than loam (9.3 Mg ha^−1^ to 14.7 Mg ha^−1^). Augustin and Cihacek^100^ determined that as sand content increases, SOC decreases. This is related to the fact that sand is composed of mainly quartz, which is considered chemically inert at normal ambient temperatures and partly inert at high temperatures and a high pH^101^. Furthermore, sand drains water relatively fast. This can reduce water availability for plants and, thereby, decrease plant productivity—which, in turn, is associated with a loss of SOC. Heavy soils with a high clay content show the lowest response ratio with respect to SOC sequestration. Our data includes only 38 values in the range of clayey soils, which were often recorded in the upper 20 cm. These were mostly collected in young reclamation trials, meaning that the small increase in SOC can be explained by the short reclamation period. Loam soils are represented by the highest number of values and show an absolute SOC increase of 12 Mg ha^−1^. Loamy soils are overall more fertile than sandy soils, so that increased plant growth leads to more biomass input, which is quickly metabolised by active microorganisms. The clay content in loamy soils is about 10% to 40% higher than in sandy soils^102^. Loamy soils sequester C through various mechanisms, including the formation of mineral-organic complexes, sorption of organic matter on clay particles, and the formation of organo-metallic compounds through humification processes^102^. The interaction of SOC with mineral surfaces is quantitatively most important. This is proven by the strong correlation of SOC stocks with clay content observed in several studies^103–105^. Finally, we highlight that although the absolute values for loam and sand are similar, the relative response for loam is more than twice as high as for sand. This shows that the capacity of loam to sequester newly introduced SOC is double that of sand.

## Conclusions

The purpose of this meta-analysis is to gain insights into the effectiveness of different recultivation methods for restoring the depleted SOC stocks of decommissioned coal mining sites. We also aim to understand their potential dependency on soil, environmental, and temporal factors. First and foremost, all investigated forms of agriculture (including livestock grazing) and forestry as well as all settings of the natural environment achieve sequestration of atmospheric C in soils, which is the desired outcome. In terms of absolute sequestration potential, forest after topsoil application ranges highest, closely followed by agriculture and forest, both without amendments. Given the small difference in SOC sequestration potential between these strategies, we suggest case-specific prioritisation based on their management-related variability and economic merits. Forest offers a more stable, low-disturbance option with little risk in terms of SOC losses. It takes a comparatively long time to yield economic gains and may compete with food production. Agriculture, on the other hand, produces short-term income and may be essential to local food security, but carries a risk of overintensification that can even result in SOC losses, as our study shows. We also demonstrate the importance of placing emphasis on pH, soil amendments, and long-term recultivation rather than focussing purely on the choice between agriculture, forest, or fallow after topsoil application. As for pH, the capacity for SOC sequestration is lowest in neutral to near-neutral conditions. Soil depths of 21–40 cm show the highest relative gains in SOC, indicating that processes shifting SOC from topsoil to deeper horizons are crucial to its long-term storage. SOC sequestration continuously rises with progressing time under recultivation. In terms of soil texture, loamy soils sequester twice as much newly introduced SOC than sandy soils. Climate-wise, tropical savanna or tropical wet and dry climate are shown to achieve the highest relative gains. However, our analysis lacks the necessary long-term studies to assess whether this impression is relativised by slower but overall greater sequestration potentials in cooler climates. It also highlights the need for more research into the recultivation potential of agroforestry. Nonetheless, our results can be applied to a wide range of environments, providing a scientific basis and action guide for political decisionmakers to achieve a CO_2_-neutral society.

## Supplementary Information

Supplementary Information.

## Data Availability

Available upon request to the corresponding author.

## Code availability

Available upon request to the corresponding author.

## References

[1] Bradford MA (2016). Managing uncertainty in soil carbon feedbacks to climate change. Nat. Clim. Change.

[2] Wang Z (2017). Human-induced erosion has offset one-third of carbon emissions from land cover change. Nat. Clim. Change.

[3] Harris NL (2012). Baseline map of carbon emissions from deforestation in tropical regions. Science.

[4] Gatti LV (2021). Amazonia as a carbon source linked to deforestation and climate change. Nature.

[5] Sanderman J, Hengl T, Fiske GJ (2017). Soil carbon debt of 12,000 years of human land use. Proc. Natl. Acad. Sci. USA.

[6] Sonter LJ, Moran CJ, Barrett DJ, Soares-Filho BS (2014). Processes of land use change in mining regions. J. Clean. Prod..

[7] Garai D, Narayana AC (2018). Land use/land cover changes in the mining area of Godavari coal fields of southern India. Egypt. J. Remote Sens. Space Sci..

[8] Ranjan R (2019). Assessing the impact of mining on deforestation in India. Resour. Policy.

[9] Dontala SP, Reddy TB, Vadde R (2015). Environmental aspects and impacts its mitigation measures of corporate coal mining. Procedia Earth Planet. Sci..

[10] FAO and UNEP. *The State of the World’s Forests 2020* (2020).

[11] Bebbington AJ (2018). Resource extraction and infrastructure threaten forest cover and community rights. Proc. Natl. Acad. Sci. USA.

[12] Golos PJ, Dixon KW (2014). Waterproofing topsoil stockpiles minimizes viability decline in the soil seed bank in an arid environment. Restor. Ecol..

[13] Maiti, S. K. & Ahirwal, J. Ecological restoration of coal mine degraded lands. In *Phytomanagement of Polluted Sites* 83–111 (Elsevier; 2019).

[14] Abdul-Kareem AW, McRae SG (1984). The effects on topsoil of long-term storage in stockpiles. Plant Soil.

[15] Ghose MK (2004). Effect of opencast mining on soil fertility. J. Sci. Ind. Res..

[16] Gupta SD, Kirby W, Pinno BD (2019). Effects of stockpiling and organic matter addition on nutrient bioavailability in reclamation soils. Soil Sci. Soc. Am. J..

[17] Ezeokoli OT, Mashigo SK, Paterson DG, Bezuidenhout CC, Adeleke RA (2019). Microbial community structure and relationship with physicochemical properties of soil stockpiles in selected South African opencast coal mines. Soil Sci. Plant Nutr..

[18] van Etten EJB, McCullough CD, Lund MA (2012). Importance of topography and topsoil selection and storage in successfully rehabilitating post-closure sand mines featuring pit lakes. Min. Technol..

[19] Kumaresan D (2017). Microbial functional capacity is preserved within engineered soil formulations used in mine site restoration. Sci. Rep..

[20] Harris JA, Birch P, Short KC (1993). The impact of storage of soils during opencast mining on the microbial community: A strategist theory interpretation. Restor. Ecol..

[21] Ganjegunte GK, Wick AF, Stahl PD, Vance GF (2009). Accumulation and composition of total organic carbon in reclaimed coal mine lands. Land Degrad. Dev..

[22] Ingram LJ, Schuman GE, Stahl PD, Spackman LK (2005). Microbial respiration and organic carbon indicate nutrient cycling recovery in reclaimed soils. Soil Sci. Soc. Am. J..

[23] Shrestha RK, Lal R (2006). Ecosystem carbon budgeting and soil carbon sequestration in reclaimed mine soil. Environ. Int..

[24] Maharaj S, Barton CD, Karathanasis TAD, Rowe HD, Rimmer SM (2007). Distinguishing, “new” from “old” organic carbon in reclaimed coal mine sites using thermogravimetry. Soil Sci..

[25] IUSS Working Group WRB. World reference base for soil resources 2014. International soil classification system for naming soils and creating legends for soil maps. Update 2015. https://www.fao.org/3/i3794en/I3794en.pdf (2015).

[26] Chaudhuri S, Pena-Yewtukhiw EM, McDonald LM, Skousen J, Sperow M (2011). Land use effects on sample size requirements for soil organic carbon stock estimations. Soil Sci..

[27] Munir MAM (2021). Interactive assessment of lignite and bamboo-biochar for geochemical speciation, modulation and uptake of Cu and other heavy metals in the copper mine tailing. Sci. Total Environ..

[28] Maus V (2020). A global-scale data set of mining areas. Sci. Data.

[29] Winkler K, Fuchs R, Rounsevell M, Herold M (2021). Global land use changes are four times greater than previously estimated. Nat. Commun..

[30] UNFCCC. Global Coal to Clean Power Statement. https://ukcop26.org/global-coal-to-clean-power-transition-statement/ (2021).

[31] IAE. Internation Energy Agency Statistics report—August 2021. https://www.iea.org/reports/coal-information-overview/production (2021).

[32] UNFCCC. End of Coal in Sight at COP26. External Press Release. https://unfccc.int/news/end-of-coal-in-sight-at-cop26 (2021).

[33] Kittel M, Goeke L, Kemfert C, Oei P-Y, von Hirschhausen C (2020). Scenarios for coal-exit in Germany—a model-based analysis and implications in the European context. Energies.

[34] SPD, Bündnis 90/Die Grünen, FDP. Mehr Fortschritt wagen – Bündnis für Freiheit, Gerechtigkeit und Nachhaltigkeit. Koalitionsvertrag 2021–2025 zwischen SPD, Bündnis 90/Die Grünen und FDP (2021).

[35] Rauner S (2020). Coal-exit health and environmental damage reductions outweigh economic impacts. Nat. Clim. Chang..

[36] Ignatyeva M, Yurak V, Pustokhina N (2020). Recultivation of post-mining disturbed land: Review of content and comparative law and feasibility study. Resources.

[37] IPCC. Restoration of Severely Degraded Lands. https://archive.ipcc.ch/ipccreports/sres/land_use/index.php?idp=199 (2000).

[38] Lal R (2015). Restoring soil quality to mitigate soil degradation. Sustainability.

[39] Wiesmeier M (2014). Carbon sequestration potential of soils in southeast Germany derived from stable soil organic carbon saturation. Glob. Change Biol..

[40] Lal R (2008). Carbon sequestration. Philos. Trans. R. Soc. Lond. Ser. B Biol. Sci..

[41] Gross A, Bromm T, Glaser B (2021). Soil organic carbon sequestration after biochar application: A global meta-analysis. Agronomy.

[42] Lal R, Negassa W, Lorenz K (2015). Carbon sequestration in soil. Curr. Opin. Environ. Sustain..

[43] Lal R, Follett RF, Stewart BA, Kimble JM (2007). Soil carbon sequestration to mitigate climate change and advance food security. Soil Sci..

[44] Rumpel C (2020). The 4p1000 initiative: Opportunities, limitations and challenges for implementing soil organic carbon sequestration as a sustainable development strategy. Ambio.

[45] Ahirwal J, Kumari S, Singh AK, Kumar A, Maiti SK (2021). Changes in soil properties and carbon fluxes following afforestation and agriculture in tropical forest. Ecol. Ind..

[46] Vindušková O, Frouz J (2013). Soil carbon accumulation after open-cast coal and oil shale mining in Northern Hemisphere: A quantitative review. Environ. Earth Sci..

[47] Nave LE, Swanston CW, Mishra U, Nadelhoffer KJ (2013). Afforestation effects on soil carbon storage in the United States: A synthesis. Soil Sci. Soc. Am. J..

[48] Sperow M (2006). Carbon sequestration potential in reclaimed mine sites in seven east-central states. J. Environ. Qual..

[49] Fox JF, Campbell JE (2010). Terrestrial carbon disturbance from mountaintop mining increases lifecycle emissions for clean coal. Environ. Sci. Technol..

[50] Allory V, Séré G, Ouvrard S (2022). A meta-analysis of carbon content and stocks in Technosols and identification of the main governing factors. Eur. J. Soil Sci..

[51] Ahirwal J, Maiti SK, Satyanarayana Reddy M (2017). Development of carbon, nitrogen and phosphate stocks of reclaimed coal mine soil within 8 years after forestation with *Prosopis juliflora* (Sw.) Dc. CATENA.

[52] Tan M (2021). Soil characteristics and microbial responses in post-mine reclamation areas in a typical resource-based city, China. J. Environ. Eng. Landsc. Manag..

[53] Peel MC, Finlayson BL, McMahon TA (2007). Updated world map of the Köppen-Geiger climate classification. Hydrol. Earth Syst. Sci..

[54] Beck HE (2018). Present and future Köppen-Geiger climate classification maps at 1-km resolution. Sci. Data.

[55] Web Plot Digitizer Version 4.4. Web based tool to extract data from plots, images, and maps. https://automeris.io/WebPlotDigitizer/.

[56] Measuring and modelling soil carbon stocks and stock changes in livestock production systems—Guidelines for assessment. Version 1. (FAO, 2019).

[57] Men, M. X., Peng, Z. P., Hao, X., Yu, Z. R. Investigation on Pedotransfer function for estimating soil bulk density in Hebei province. *Chinese J. Soil Sci.* **1**(20) (2008).

[58] Manrique LA, Jones CA (1991). Bulk Density of Soils in Relation to Soil Physical and Chemical Properties. Soil Sci. Soc. Am. J..

[59] Gross A, Glaser B (2021). Meta-analysis on how manure application changes soil organic carbon storage. Sci. Rep..

[60] Hübner, R., Kühnel, A., Jie, L., Dettmann, H., Wang, W. & Wiesmeier, M. Soil carbon sequestration by agroforestry systems in China: A meta-analysis. *Agric. Ecosyst. Environ.*10.1016/j.agee.2021.107437 (2021).

[61] Chaudhuri S, Pena-Yewtukhiw EM, McDonald LM, Skousen J, Sperow M (2012). Early C sequestration rate changes for reclaimed minesoils. Soil Sci..

[62] Wick AF, Ingram LJ, Stahl PD (2009). Aggregate and organic matter dynamics in reclaimed soils as indicated by stable carbon isotopes. Soil Biol. Biochem..

[63] Leal ODA (2015). Organic matter fractions and quality of the surface layer of a constructed and vegetated soil after coal mining. I—Humic substances and chemical characterization. Rev. Bras. Ciênc. Solo.

[64] Leal ODA (2016). Initial recovery of organic matter of a grass-covered constructed soil after coal mining. Rev. Bras. Ciênc. Solo.

[65] Yuan Y (2017). Soil organic carbon and nitrogen pools in reclaimed mine soils under forest and cropland ecosystems in the Loess Plateau, China. Ecol. Eng..

[66] Koricheva J, Gurevitch J, Mengersen K (2013). Handbook of Meta-analysis in Ecology and Evolution.

[67] Gurevitch J, Koricheva J, Nakagawa S, Stewart G (2018). Meta-analysis and the science of research synthesis. Nature.

[68] Han P, Zhang W, Wang G, Sun W, Huang Y (2016). Changes in soil organic carbon in croplands subjected to fertilizer management: a global meta-analysis. Sci. Rep..

[69] Guo LB, Gifford RM (2002). Soil carbon stocks and land use change: A meta analysis. Glob. Change Biol..

[70] Johnson DW, Curtis PS (2001). Effects of forest management on soil C and N storage: Meta analysis. For. Ecol. Manag..

[71] Qin W, Hu C, Oenema O (2015). Soil mulching significantly enhances yields and water and nitrogen use efficiencies of maize and wheat: A meta-analysis. Sci. Rep..

[72] The R Project for Statistical Computing. Integrated Development Enviroment for R. Version 4.1.0. http://www.rstudio.com/ (2021).

[73] Lal R (2005). Forest soils and carbon sequestration. For. Ecol. Manage..

[74] Akala VA, Lal R (2001). Soil organic carbon pools and sequestration rates in reclaimed minesoils in Ohio. J. Environ. Qual..

[75] Shukla MK, Lal R (2005). Temporal changes in soil organic carbon concentration and stocks in reclaimed minesoils of Southeastern Ohio. Soil Sci..

[76] Luo Z, Feng W, Luo Y, Baldock J, Wang E (2017). Soil organic carbon dynamics jointly controlled by climate, carbon inputs, soil properties and soil carbon fractions. Glob. Change Biol..

[77] Wiesmeier M (2019). Soil organic carbon storage as a key function of soils—A review of drivers and indicators at various scales. Geoderma.

[78] Malik AA (2018). Land use driven change in soil pH affects microbial carbon cycling processes. Nat. Commun..

[79] Carvalhais N (2014). Global covariation of carbon turnover times with climate in terrestrial ecosystems. Nature.

[80] Ivezić V, Lorenz K, Lal R (2022). Soil organic carbon in alley cropping systems: A meta-analysis. Sustainability.

[81] Ma Z, Chen HYH, Bork EW, Carlyle CN, Chang SX (2020). Carbon accumulation in agroforestry systems is affected by tree species diversity, age and regional climate: A global meta-analysis. Glob. Ecol. Biogeogr..

[82] Bradford JB, Kastendick DN (2010). Age-related patterns of forest complexity and carbon storage in pine and aspen–birch ecosystems of northern Minnesota, USA. Can. J. For. Res..

[83] Bárcena TG (2014). Soil carbon stock change following afforestation in Northern Europe: A meta-analysis. Glob. Change Biol..

[84] Lorenz M, Thiele-Bruhn S (2019). Tree species affect soil organic matter stocks and stoichiometry in interaction with soil microbiota. Geoderma.

[85] Nakagami K (2009). Soil carbon stock in typical grasslands in Japan. Grassland Sci..

[86] Čížková B, Woś B, Pietrzykowski M, Frouz J (2018). Development of soil chemical and microbial properties in reclaimed and unreclaimed grasslands in heaps after opencast lignite mining. Ecol. Eng..

[87] Carolan R, Fornara DA (2016). Soil carbon cycling and storage along a chronosequence of re-seeded grasslands: Do soil carbon stocks increase with grassland age?. Agric. Ecosyst. Environ..

[88] Reichel R, Hänsch M, Brüggemann N (2017). Indication of rapid soil food web recovery by nematode-derived indices in restored agricultural soil after open-cast lignite mining. Soil Biol. Biochem..

[89] Angers DA, Eriksen-Hamel NS (2008). Full-inversion tillage and organic carbon distribution in soil profiles: A meta-analysis. Soil Sci. Soc. Am. J..

[90] Don, A. *et al.* Die 4-Promille-Initiative “Böden für Ernährungssicherung und Klima – Wissenschaftliche Bewertung und Diskussion möglicher Beiträge in Deutschland. Thuenen Working Paper 112 (2018).

[91] Shi L, Feng W, Xu J, Kuzyakov Y (2018). Agroforestry systems: Meta-analysis of soil carbon stocks, sequestration processes, and future potentials. Land Degrad. Dev..

[92] Muchane, M. N., Sileshi, G. W., Gripenberg, S., Jonsson, M., Pumariño, L., Barrios, E. Agroforestry boosts soil health in the humid and sub-humid tropics: A meta-analysis. *Agric. Ecosyst. Environ.*10.1016/j.agee.2020.106899 (2020).

[93] Mayer, S. *et al.* Soil organic carbon sequestration in temperate agroforestry systems – A meta-analysis. *Agric. Ecosyst. Environ.* **323**, 107689; 10.1016/j.agee.2021.107689 (2022).

[94] Luo Z, Feng W, Luo Y, Baldock J, Wang E (2017). Soil organic carbon dynamics jointly controlled by climate, carbon inputs, soil properties and soil carbon fractions. Global Change Biology.

[95] Kemmit S, Wright D, Goulding K, Jones D (2006). pH regulation of carbon and nitrogen dynamics in two agricultural soils. Soil Biol. Biochem..

[96] Rousk J, Brookes PC, Bååth E (2009). Contrasting soil pH effects on fungal and bacterial growth suggest functional redundancy in carbon mineralization. Appl. Environ. Microbiol..

[97] Xiao D (2018). Soil organic carbon mineralization with fresh organic substrate and inorganic carbon additions in a red soil is controlled by fungal diversity along a pH gradient. Geoderma.

[98] Ahirwal J, Kumar A, Pietrzykowski M, Maiti SK (2018). Reclamation of coal mine spoil and its effect on Technosol quality and carbon sequestration: A case study from India. Environ. Sci. Pollut. Res. Int..

[99] Demyan MS, Smeck N (2022). Chemical, physical-temporal and spatial changes in 25-year-old mine soils in Southeast Ohio. Land Degrad. Dev..

[100] Augustin C, Cihacek LJ (2016). Relationships between soil carbon and soil texture in the Northern Great Plains. Soil Sci..

[101] Lin R-S, Wang X-Y, Zhang G-Y (2018). Effects of quartz powder on the microstructure and key properties of cement paste. Sustainability.

[102] Kome GK, Enang RK, Tabi FO, Yerima BPK (2019). Influence of clay minerals on some soil fertility attributes: A review. OJSS.

[103] Arrouays D, Saby N, Walter C, Lemercier B, Schvartz C (2006). Relationships between particle-size distribution and organic carbon in French arable topsoils. Soil Use Manag..

[104] Zinn YL, Lal R, Bigham JM, Resck DVS (2007). Edaphic controls on soil organic carbon retention in the Brazilian Cerrado: Texture and mineralogy. Soil Sci. Soc. Am. J..

[105] Kaiser K, Guggenberger G (2000). The role of DOM sorption to mineral surfaces in the preservation of organic matter in soils. Org. Geochem..

