# Supplementary material for: Effects of recultivation on soil organic carbon sequestration in abandoned coal mining sites: a meta-analysis
Source: Sci Rep. 2022 Nov 22;12:20090. doi: 10.1038/s41598-022-22937-z (PMC9684481; doi:10.1038/s41598-022-22937-z)
Supplement: Supplementary file 1 — Supplementary Information. [file 41598_2022_22937_MOESM1_ESM.docx]

Baier, C.; Modersohn, A.; Jalowy, F.; Glaser, B.; and Gross, A.: Effects of recultivation on soil organic carbon sequestration in abandoned coal mining sites: a meta-analysis.

**Supplementary Material**

**Breakdown of individual substrategies within the overall categories “agriculture”, “forest”, and “topsoil”:**

The individual categories consisted of: (i) agriculture without amendments, (ii) agriculture with fertiliser, (iii) agriculture after topsoil application, (iv) agriculture after topsoil and fertiliser application, (v) agriculture after liming, topsoil, and fertiliser application, (vi) agroforestry with fertiliser, (vii) forest without amendments, (viii) forest after topsoil application, (ix) forest with fertiliser, (x) forest with liming, (xi) forest after topsoil and fertiliser application, (xii) fallow after topsoil application, and (xiii) fallow after topsoil and fertiliser application.

**Results of the statistical analysis:**

The regression analysis investigating whether the sequestration rate and stock difference are dependent on time revealed a significant dependence in both cases (sequestration rate: r²=0.01184, p=0.01679; difference in SOC stocks: r²=0.1104 and p=0.000000000005835).

The Kruskal-Wallis-Test followed by the Dunn-Bonferroni-Test to determine significant differences between the time under recultivation and climate or recultivation strategy revealed the following results:

**Tab. S1:** Results of the Kruskal-Wallis multiple comparison test followed by the Dunn-Bonferroni post-hoc test. Climate zones are compared and results are presented as p-values. Climate zones are considered significantly different if p<0.05.


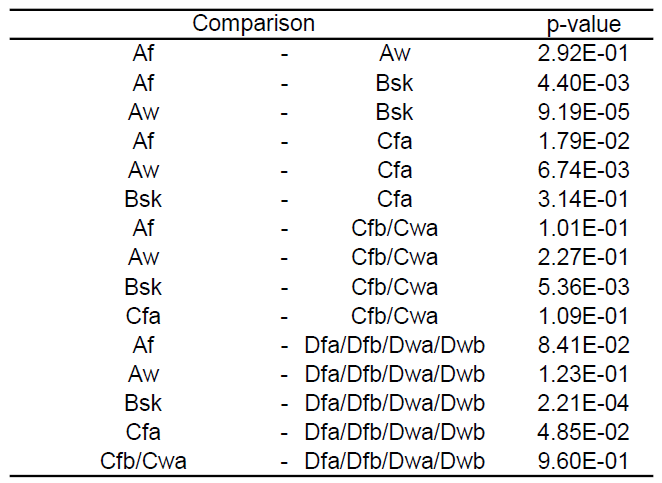


**Tab. S2:** Results of the Kruskal-Wallis multiple comparison test followed by the Dunn-Bonferroni post-hoc test. Recultivation strategies are compared and results are presented as p-values. Recultivation strategies are considered significantly different if p<0.05.


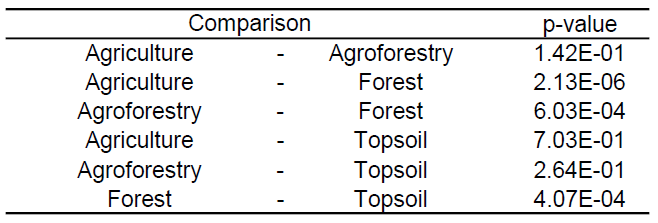


**Tab. S3:** Studies, number of observations, and types of experimental controls included in this meta-analysis. For better visualisation, “-“ stands for “not accurate”.

| Study | no. of pairwise data entries included in this meta-analysis | satisfactory control | control = SOC value after 1 year of cultivation because study included no control | control = SOC value after 1 year of cultivation because control was nearby undisturbed ecosystem |
| --- | --- | --- | --- | --- |
| Acton et al. (2011) ^1^ | 5 | accurate | **-** | **-** |
| Adeli et al. (2013) ^2^ | 12 | accurate | **-** | **-** |
| Adeli et al. (2019) ^3^ | 8 | accurate | **-** | **-** |
| Agus et al. (2016) ^4^ | 5 | accurate | **-** | **-** |
| Ahirwal et al. (2017) ^5^ | 21 | **-** | **-** | accurate |
| Ahirwal et al. (2018) ^6^ | 4 | accurate | **-** | **-** |
| Ahirwal and Maiti (2018) ^7^ | 1 | accurate | **-** | **-** |
| Akala and Lal (2001) ^8^ | 34 | accurate | **-** | **-** |
| Bodlák et al. (2012) ^9^ | 5 | **-** | accurate | **-** |
| Brooks et al. (2019) ^10^ | 8 | **-** | **-** | accurate |
| Chatterjee et al. (2009) ^11^ | 4 | **-** | **-** | accurate |
| Chaudhuri et al. (2011) ^12^ | 2 | **-** | accurate | **-** |
| Chaudhuri et al. (2012) ^13^ | 6 | **-** | accurate | **-** |
| Clayton et al. (2021) ^14^ | 9 | **-** | accurate | **-** |
| Das and Maiti (2016) ^15^ | 5 | accurate | **-** | **-** |
| Demyan and Smeck (2022) ^16^ | 1 | accurate | **-** | **-** |
| Filcheva et al. (2000) ^17^ | 6 | accurate | **-** | **-** |
| Fu et al. (2010) ^18^ | 16 | accurate | **-** | **-** |
| Ganjegunte et al. (2009) ^19^ | 4 | accurate | **-** | **-** |
| Greinert et al. (2018) ^20^ | 9 | accurate | **-** | **-** |
| Haigh et al. (2020) ^21^ | 1 | accurate | **-** | **-** |
| Jacinthe and Lal (2009) ^22^ | 6 | **-** | **-** | accurate |
| Jambhulkar and Kumar (2019) ^23^ | 3 | accurate | **-** | **-** |
| Kanzler et al. (2021) ^24^ | 15 | accurate | **-** | **-** |
| Leal et al. (2015) ^25^ | 4 | accurate | **-** | **-** |
| Leal et al. (2016) ^26^ | 6 | accurate | **-** | **-** |
| Li et al. (2015) ^27^ | 5 | **-** | accurate | **-** |
| Li et al. (2019) ^28^ | 12 | accurate | **-** | **-** |
| Littlefield et al. (2013) ^29^ | 2 | **-** | accurate | **-** |
| Lorenz and Thiele-Bruhn (2019) ^30^ | 14 | accurate | **-** | **-** |
| Maharaj et al. (2007) ^31^ | 1 | accurate | **-** | **-** |
| Mukhopadhyay et al. (2016) ^32^ | 4 | accurate | **-** | **-** |
| Osei‐Tutu et al. (2018) ^33^ | 2 | accurate | **-** | **-** |
| Pihlap et al. (2019) ^34^ | 5 | accurate | **-** | **-** |
| Placek-Lapaj et al. (2019) ^35^ | 12 | accurate | **-** | **-** |
| Qiu et al. (2019) ^36^ | 4 | **-** | accurate | **-** |
| Reichel et al. (2017) ^37^ | 3 | accurate | **-** | **-** |
| Rodionov et al. (2012) ^38^ | 12 | **-** | accurate | **-** |
| Růžek et al. (2003) ^39^ | 10 | accurate | **-** | **-** |
| Shrestha and Lal (2010) ^40^ | 36 | **-** | accurate | **-** |
| Shukla and Lal (2005) ^41^ | 10 | **-** | **-** | accurate |
| Tan et al. (2021) ^42^ | 5 | accurate | **-** | **-** |
| Vlachodimos et al. (2013) ^43^ | 4 | accurate | **-** | **-** |
| Wang et al. (2021) ^44^ | 4 | accurate | **-** | **-** |
| Wick et al. (2009) ^45^ | 2 | accurate | **-** | **-** |
| Wick et al. (2009) ^46^ | 3 | accurate | **-** | **-** |
| Yang et al. (2018) ^47^ | 4 | accurate | **-** | **-** |
| Yin et al. (2016) ^48^ | 8 | accurate | **-** | **-** |
| Yuan et al. (2017) ^49^ | 9 | accurate | **-** | **-** |
| Yuan et al. (2018) ^50^ | 12 | accurate | **-** | **-** |
| Zhang et al. (2020) ^51^ | 16 | accurate | **-** | **-** |


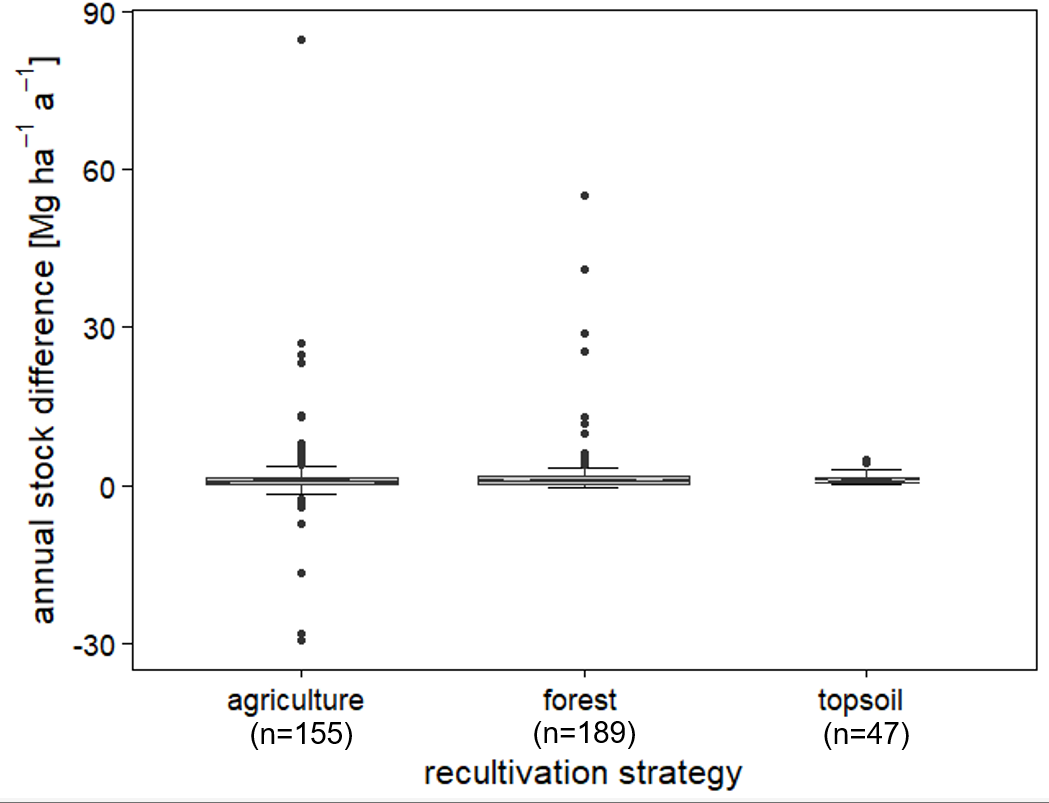


**Fig. S1: Original version of figure 2, i. e. inluding all outliers.** It shows the annual amount of SOC sequestered by mine soils [Mg ha^-1^ a^-1^] that were recultivated employing the three main recultivation strategies (i) agriculture (incl. agriculture, agriculture with fertiliser, agriculture with topsoil, agriculture with topsoil and fertilizer, and agriculture with liming, topsoil, and fertiliser), (ii) forest (incl. forest, forest with topsoil, forest with liming, and forest with fertiliser), and (iii) topsoil (incl. topsoil by itself and topsoil with fertiliser). Agroforestry was omitted from this figure, as it was decidedly underrepresented with one single study containing 15 values. Each box contains the middle 50% of the data of a category. The mean of the data is shown as a vertical solid line within the box. The whiskers indicate the lower and upper quartile of the data and are limited to 1.5 times the interquartile range.


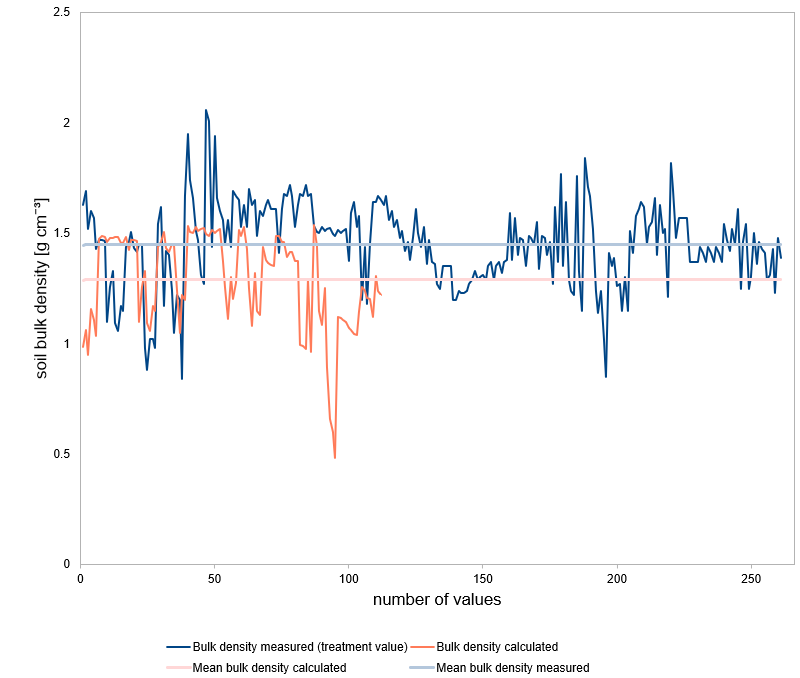


**Fig. S2:** Visualisation of the deviation between the bulk densities that were reported by the original studies (i. .e measured) and those we calculated using pedotransfer functions.

**References**

1. Acton, P. M. *et al.* Role of soil health in maintaining environmental sustainability of surface coal mining. *Environmental science & technology* **45,** 10265–10272; 10.1021/es202764q (2011).

2. Adeli, A. *et al.* Age Chronosequence Effects on Restoration Quality of Reclaimed Coal Mine Soils in Mississippi Agroecosystems. *Soil Science* **178,** 335–343; 10.1097/SS.0b013e3182a79e37 (2013).

3. Adeli, A., Brooks, J. P., Read, J. J., McGrew, R. & Jenkins, J. N. Post-reclamation Age Effects on Soil Physical Properties and Microbial Activity Under Forest and Pasture Ecosystems. *Communications in Soil Science and Plant Analysis* **50,** 20–34; 10.1080/00103624.2018.1546868 (2019).

4. Agus, C., Putra, P. B., Faridah, E., Wulandari, D. & Napitupulu, R. R.P. Organic Carbon Stock and their Dynamics in Rehabilitation Ecosystem Areas of Post Open Coal Mining at Tropical Region. *Procedia Engineering* **159,** 329–337; 10.1016/j.proeng.2016.08.201 (2016).

5. Ahirwal, J., Maiti, S. K. & Satyanarayana Reddy, M. Development of carbon, nitrogen and phosphate stocks of reclaimed coal mine soil within 8 years after forestation with Prosopis juliflora (Sw.) Dc. *CATENA* **156,** 42–50; 10.1016/j.catena.2017.03.019 (2017).

6. Ahirwal, J., Kumar, A., Pietrzykowski, M. & Maiti, S. K. Reclamation of coal mine spoil and its effect on Technosol quality and carbon sequestration: a case study from India. *Environmental science and pollution research international* **25,** 27992–28003; 10.1007/s11356-018-2789-1 (2018).

7. Ahirwal, J. & Maiti, S. K. Assessment of soil carbon pool, carbon sequestration and soil CO2 flux in unreclaimed and reclaimed coal mine spoils. *Environ Earth Sci* **77**; 10.1007/s12665-017-7185-5 (2018).

8. Akala, V. A. & Lal, R. Soil organic carbon pools and sequestration rates in reclaimed minesoils in Ohio. *Journal of environmental quality* **30,** 2098–2104; 10.2134/jeq2001.2098 (2001).

9. Bodlák, L. *et al.* SOC content—An appropriate tool for evaluating the soil quality in a reclaimed post-mining landscape. *Ecological Engineering* **43,** 53–59; 10.1016/j.ecoleng.2011.07.013 (2012).

10. Brooks, J. P. *et al.* Bacterial Community Structure Recovery in Reclaimed Coal Mined Soil under Two Vegetative Regimes. *Journal of environmental quality* **48,** 1029–1037; 10.2134/jeq2018.09.0349 (2019).

11. Chatterjee, A., Lal, R., Shrestha, R. K. & Ussiri, D.A.N. Soil carbon pools of reclaimed minesoils under grass and forest landuses. *Land Degrad. Dev.* **20,** 300–307; 10.1002/ldr.916 (2009).

12. Chaudhuri, S., Pena-Yewtukhiw, E. M., McDonald, L. M., Skousen, J. & Sperow, M. Land Use Effects on Sample Size Requirements for Soil Organic Carbon Stock Estimations. *Soil Science* **176,** 110–114; 10.1097/SS.0b013e31820a0fe2 (2011).

13. Chaudhuri, S., Pena-Yewtukhiw, E. M., McDonald, L. M., Skousen, J. & Sperow, M. Early C Sequestration Rate Changes for Reclaimed Minesoils. *Soil Science* **177,** 443–450; 10.1097/SS.0b013e318254494d (2012).

14. Clayton, J., Lemanski, K. & Bonkowski, M. Shifts in soil microbial stoichiometry and metabolic quotient provide evidence for a critical tipping point at 1% soil organic carbon in an agricultural post-mining chronosequence. *Biol Fertil Soils* **57,** 435–446; 10.1007/s00374-020-01532-2 (2021).

15. Das, R. & Maiti, S. K. Importance of carbon fractionation for the estimation of carbon sequestration in reclaimed coalmine soils—A case study from Jharia coalfields, Jharkhand, India. *Ecological Engineering* **90,** 135–140; 10.1016/j.ecoleng.2016.01.025 (2016).

16. Demyan, M. S. & Smeck, N. Chemical, physical‐temporal and spatial changes in 25‐year‐old mine soils in Southeast Ohio. *Land Degrad. Dev.* **33,** 294–307; 10.1002/ldr.4150 (2022).

17. Filcheva, E., Noustorova, M., Gentcheva-Kostadinova, S. & Haigh, M.J. Organic accumulation and microbial action in surface coal-mine spoils, Pernik, Bulgaria. *Ecological Engineering* **15,** 1–15; 10.1016/S0925-8574(99)00008-7 (2000).

18. Fu, Y., Lin, C., Ma, J. & Zhu, T. Effects of plant types on physico-chemical properties of reclaimed mining soil in Inner Mongolia, China. *Chin. Geogr. Sci.* **20,** 309–317; 10.1007/s11769-010-0403-7 (2010).

19. Ganjegunte, G. K., Wick, A. F., Stahl, P. D. & Vance, G. F. Accumulation and composition of total organic carbon in reclaimed coal mine lands. *Land Degrad. Dev.* **20,** 156–175; 10.1002/ldr.889 (2009).

20. Greinert, A., Drab, M. & Śliwińska, A. STORAGE CAPACITY OF ORGANIC CARBON IN THE RECLAIMED POST-MINING TECHNOSOLS. *Environment Protection Engineering* **44** (2018).

21. Haigh, M. *et al.* Successful Ecological Regeneration of Opencast Coal Mine Spoils through Forestation: From Cradle to Grove. *Minerals* **10,** 461; 10.3390/min10050461 (2020).

22. Jacinthe, P.-A. & Lal, R. Tillage Effects on Carbon Sequestration and Microbial Biomass in Reclaimed Farmland Soils of Southwestern Indiana. *Soil Sci. Soc. Am. J.* **73,** 605–613; 10.2136/sssaj2008.0156 (2009).

23. Jambhulkar, H. P. & Kumar, M. S. Eco-restoration approach for mine spoil overburden dump through biotechnological route. *Environmental monitoring and assessment* **191,** 772; 10.1007/s10661-019-7873-6 (2019).

24. Kanzler, M., Böhm, C. & Freese, D. The development of soil organic carbon under young black locust (Robinia pseudoacacia L.) trees at a post-mining landscape in eastern Germany. *New Forests* **52,** 47–68; 10.1007/s11056-020-09779-1 (2021).

25. Leal, O. d. A. *et al.* Organic Matter Fractions and Quality of the Surface Layer of a Constructed and Vegetated Soil After Coal Mining. I - Humic Substances and Chemical Characterization. *Rev. Bras. Ciênc. Solo* **39,** 886–894; 10.1590/01000683rbcs20140783 (2015).

26. Leal, O. d. A. *et al.* Initial Recovery of Organic Matter of a Grass-Covered Constructed Soil after Coal Mining. *Rev. Bras. Ciênc. Solo* **40**; 10.1590/18069657rbcs20150384 (2016).

27. Li, J., Zhou, X., Yan, J., Li, H. & He, J. Effects of regenerating vegetation on soil enzyme activity and microbial structure in reclaimed soils on a surface coal mine site. *Applied Soil Ecology* **87,** 56–62; 10.1016/j.apsoil.2014.11.010 (2015).

28. Li, J. *et al.* Effects of fertilization and straw return methods on the soil carbon pool and CO2 emission in a reclaimed mine spoil in Shanxi Province, China. *Soil and Tillage Research* **195,** 104361; 10.1016/j.still.2019.104361 (2019).

29. Littlefield, T., Barton, C., Arthur, M. & Coyne, M. Factors controlling carbon distribution on reforested minelands and regenerating clearcuts in Appalachia, USA. *The Science of the total environment* **465,** 240–247; 10.1016/j.scitotenv.2012.12.029 (2013).

30. Lorenz, M. & Thiele-Bruhn, S. Tree species affect soil organic matter stocks and stoichiometry in interaction with soil microbiota. *Geoderma* **353,** 35–46; 10.1016/j.geoderma.2019.06.021 (2019).

31. Maharaj, S., Barton, C. D., Karathanasis, T. A. D., Rowe, H. D. & Rimmer, S. M. Distinguishing “New” from “Old” Organic Carbon in Reclaimed Coal Mine Sites Using Thermogravimetry. *Soil Science* **172,** 302–312; 10.1097/SS.0b013e3180314702 (2007).

32. Mukhopadhyay, S. *et al.* Soil quality index for evaluation of reclaimed coal mine spoil. *The Science of the total environment* **542,** 540–550; 10.1016/j.scitotenv.2015.10.035 (2016).

33. Osei‐Tutu, G., Abunyewa, A. A., Dawoe, E. K., Agbenyega, O. & Barnes, R. V. Effect of multipurpose trees and shrubs on degraded mined‐out soil in a semi‐deciduous forest zone of West Africa. *Land Degrad. Dev.* **29,** 3432–3439; 10.1002/ldr.3110 (2018).

34. Pihlap, E. *et al.* Initial soil formation in an agriculturally reclaimed open-cast mining area - the role of management and loess parent material. *Soil and Tillage Research* **191,** 224–237; 10.1016/j.still.2019.03.023 (2019).

35. Placek-Lapaj, A. *et al.* Post - Mining soil as carbon storehouse under polish conditions. *Journal of environmental management* **238,** 307–314; 10.1016/j.jenvman.2019.03.005 (2019).

36. Qiu, L. *et al.* Arbuscular mycorrhizal fungi ameliorate the chemical properties and enzyme activities of rhizosphere soil in reclaimed mining subsidence in northwestern China. *J. Arid Land* **11,** 135–147; 10.1007/s40333-018-0019-9 (2019).

37. Reichel, R., Hänsch, M. & Brüggemann, N. Indication of rapid soil food web recovery by nematode-derived indices in restored agricultural soil after open-cast lignite mining. *Soil Biology and Biochemistry* **115,** 261–264; 10.1016/j.soilbio.2017.08.020 (2017).

38. Rodionov, A. *et al.* Impacts of Soil Additives on Crop Yield and C-Sequestration in Post Mine Substrates of Lusatia, Germany. *Pedosphere* **22,** 343–350; 10.1016/S1002-0160(12)60021-6 (2012).

39. Růžek, L., Voříšek, K., Vráblíková, J., Strnadová, S. & Vráblík, P. Chemical and biological characteristics of reclaimed soils in the Most ŕegion (Czecg Reooublic). *Plant Soil Environ.* **49,** 346–351; 10.17221/4135-PSE (2003).

40. Shrestha, R. K. & Lal, R. Carbon and nitrogen pools in reclaimed land under forest and pasture ecosystems in Ohio, USA. *Geoderma* **157,** 196–205; 10.1016/j.geoderma.2010.04.013 (2010).

41. Shukla, M. K. & Lal, R. TEMPORAL CHANGES IN SOIL ORGANIC CARBON CONCENTRATION AND STOCKS IN RECLAIMED MINESOILS OF SOUTHEASTERN OHIO. *Soil Science* **170,** 1013–1021; 10.1097/01.ss.0000187354.62481.91 (2005).

42. Tan, M. *et al.* SOIL CHARACTERISTICS AND MICROBIAL RESPONSES IN POST-MINE RECLAMATION AREAS IN A TYPICAL RESOURCE-BASED CITY, CHINA. *Journal of Environmental Engineering and Landscape Management* **29,** 273–286; 10.3846/jeelm.2021.15138 (2021).

43. Vlachodimos, K., Papatheodorou, E. M., Diamantopoulos, J. & Monokrousos, N. Assessment of Robinia pseudoacacia cultivations as a restoration strategy for reclaimed mine spoil heaps. *Environmental monitoring and assessment* **185,** 6921–6932; 10.1007/s10661-013-3075-9 (2013).

44. Wang, K. *et al.* Shifts in composition and function of soil fungal communities and edaphic properties during the reclamation chronosequence of an open-cast coal mining dump. *The Science of the total environment* **767,** 144465; 10.1016/j.scitotenv.2020.144465 (2021).

45. Wick, A. F., Ingram, L. J. & Stahl, P. D. Aggregate and organic matter dynamics in reclaimed soils as indicated by stable carbon isotopes. *Soil Biology and Biochemistry* **41,** 201–209; 10.1016/j.soilbio.2008.09.012 (2009).

46. Wick, A. F., Stahl, P. D. & Ingram, L. J. Aggregate-Associated Carbon and Nitrogen in Reclaimed Sandy Loam Soils. *Soil Sci. Soc. Am. J.* **73,** 1852–1860; 10.2136/sssaj2008.0011 (2009).

47. Yang, N., Ji, L., Salahuddin, Yang, Y. & Yang, L. The influence of tree species on soil properties and microbial communities following afforestation of abandoned land in northeast China. *European Journal of Soil Biology* **85,** 73–78; 10.1016/j.ejsobi.2018.01.003 (2018).

48. Yin, N., Zhang, Z., Wang, L. & Qian, K. Variations in organic carbon, aggregation, and enzyme activities of gangue-fly ash-reconstructed soils with sludge and arbuscular mycorrhizal fungi during 6-year reclamation. *Environmental science and pollution research international* **23,** 17840–17849; 10.1007/s11356-016-6941-5 (2016).

49. Yuan, Y. *et al.* Soil organic carbon and nitrogen pools in reclaimed mine soils under forest and cropland ecosystems in the Loess Plateau, China. *Ecological Engineering* **102,** 137–144; 10.1016/j.ecoleng.2017.01.028 (2017).

50. Yuan, Y., Zhao, Z., Li, X., Wang, Y. & Bai, Z. Characteristics of labile organic carbon fractions in reclaimed mine soils: Evidence from three reclaimed forests in the Pingshuo opencast coal mine, China. *The Science of the total environment* **613-614,** 1196–1206; 10.1016/j.scitotenv.2017.09.170 (2018).

51. Zhang, P. P. *et al.* Revegetation pattern affecting accumulation of organic carbon and total nitrogen in reclaimed mine soils. *PeerJ* **8,** e8563; 10.7717/peerj.8563 (2020).
